# Numerical study of magneto convective ag (silver) graphene oxide (GO) hybrid nanofluid in a square enclosure with hot and cold slits and internal heat generation/absorption

**DOI:** 10.1038/s41598-024-76233-z

**Published:** 2024-10-22

**Authors:** Elayaraja Rajenderan, V. Ramachandra Prasad

**Affiliations:** Department of Mathematics, School of Advances Sciences, Vellore Institute of Technology, Vellore, 632014 India

**Keywords:** Mixed convection, Water hybrid nanofluid, MAC numerical technique, Square enclosure, Applied mathematics, Fluid dynamics

## Abstract

Energy transmission is widely used in various engineering industries. In recent times, the utilization of hybrid nanofluids has become one of the most popular choices in various industrial fields to increase thermal performance and enhance power generation, entropy reduction, solar collectors, and solar systems. Motivated by this wide range of applications, the present article explores the mixed convection flow and heat transfer of magnetohydrodynamic $\:Ag$ (*Silver*) and $\:GO$ (*Graphene*) nanofluids hybrid nanofluids in a square enclosure with heat generation/absorption by using the MAC method. The vertical walls of the enclosure are assumed to be adiabatic. The horizontal walls are also assumed adiabatic except for the center portion of the top and bottom walls of the cavity. The center portion of the horizontal upper wall is maintained as a cold is $\:{(T}_{c})$and the lower wall is maintained as hot $\:\left({T}_{h}\right)$. The dimension equations are transformed into dimensionless form and then discretized and solved with the finite difference Marker and cell (MAC) method. Numerical modelling is implemented, by changing Richardson number $\:\left(Ri\right)$, The results are located graphically using MATLAB software. The Nusselt number graph was displayed for the Reynolds number (Re), Richardson number$\:\:\left(Ri\right)$, and Hartmann number $\:\left(Ha\right)$. The findings show that enhancing the values of the Richardson number and Reynolds number enhances the Nusselt number values except for the Hartmann number. The findings indicate that the combination of the new model is very good at predicting thermal conductivity and correlates experimental results well. The augmenting strength of magnetic force diminishes fluid flow. Developing the coefficients for the heat source and sink improves energy transmission and heat transfer enhancement. Hybrid nanofluids like $\:Ag-GO$ enhance heat transfer and efficiency. They improve cooling in heat exchangers, radiators, and electronics, boost solar energy systems, aid in cancer treatment and drug delivery, enhance geothermal and wind turbine efficiency, and improve manufacturing processes. Overall, they optimize thermal management in various applications.

## Introduction

Nanofluids are considered higher-quality fluids than regular fluids in thermal systems. Thermal control of components for semiconductor technology is a rigorous execution to sustain their base temperature in a secure and optimal range. Hybrid nanofluids are widely used in many applications, including heat sink systems, because of their exceptional features. The majority of research has found that using hybrid nanofluids rather than regular fluids outcomes in the development of heat transfer in heat sinks. These fluids are extremely useful in a wide range of heat transfer-related applications, including microelectronics, hybrid engines, vehicle heating and cooling, heat exchangers, home freezers, and other applications. The efficiency of HT fluids is determined by thermal conductivity, viscosity, density, volume fraction, and heating efficiency, which determines the path of energy exchange in heat transfer systems. The HT fluids have inadequate thermal conductivity, and this issue is solved using different nano-particles. Recent developments in the study have made it easy for nanoparticles to permeate into commonly used heat-transfer fluids involving water, oil and ethylene glycol $\:\left({C}_{2}{H}_{6}{O}_{2}\right)$, generating a new kind of fluid with improved heat-transfer efficiency. A liquid and a combination of suspended nanoparticles create a nanofluid Aluminium oxide$\:\left({Al}_{2}{O}_{3}\right)$, silicon dioxide $\:\left(\text{S}\text{i}{O}_{2}\right)$, Silver $\:\left(\text{A}\text{g}\right),$ Copper $\:\left(\text{C}\text{u}\right)$, Titanium dioxide $\:\left(Ti{O}_{2}\right)\:$and Magnesium oxide $\:\left(\text{M}\text{g}\text{O}\right)$ are just a few of the metals that make up the nanoparticles. In recent years, a novel type of heat transfer medium known as nanofluids has emerged. Nanofluids are composed of basic fluids and nanoparticles. Eventually, it can be noticed that the hybrid nanofluids generally improve heat transfer, reducing the volume of heat exchangers, saving energy, and reducing water use and industrial waste.

Tahar Tayebi and Ali J. Chamkha^1^ analyzed the presence of Hartmann number on entropy generation and free convection in a square chamber having a conductive wavy solid block filled with hybrid nanofluids. Rizwan Ul Haq et al.^2^ utilized the numerical finite element approach to analyze the free convection velocity and heat transfer properties of the MHD fluid within the corrugated rectangular chamber. Rahman et al.^3^ examination of steady free convection flow within a inside square porous container with nanofluids saturated permeable chamber applied the Buongiorno model with magnetic force. Husain et al.^4^ mathematically investigate the mixed convective flow in a lid-driven chamber filled with nanofluids with the existence of an enclosure inclination on heat transfer. An analysis of how a magnetic force, entropy generation, and viscous dispersion affect the irreversibility of natural Casson fluid convection cavity inclination in a porous cavity was studied by^5^. The Prandtl number effect on transient mixed convection and entropy generation in a square chamber heated downwards was discussed in^6^. Gorasn et al.^7^ studied the effect of Hartmann number and internal heat generation effects on rectangular cavity in a porous medium. Partiban and Prasad^8^ studied MHD hybrid nanofluid flow in the differentially heated cavity with heat generation/absorption by applying the Lattice Boltzmann method (LBM). The $\:Cu-A{l}_{2}{O}_{3}\:/$ water hybrid nanofluids stored in a square chamber that is oscillating, creating mixed convection flow were studied by^9^. Arifuzzaman and Uddin^10^ considered a heat-producing chemical reaction and a hydromagnetic field through alumina-water nanofluids in a square vessel.

Free convection of hybrid nanofluids in porous material under the presence of an inclined periodic magnetic effect was examined^11^. A vented square enclosure with aiding and opposing flows of MWCNT-water nanofluids was investigated in the presence of an inclined magnetic force^12^. Analyzed the performance of a hybrid $\:{\text{A}\text{l}}_{2}{\text{O}}_{3}-\text{S}\text{i}{\text{O}}_{2}$/water nanofluids for hot transfer using theoretical and experimental thermal conductivity models^13^. Yildiz et al.^14^ computed a two-phase model mixed convection hybrid $\:{\text{A}\text{l}}_{2}{\text{O}}_{3}-\text{C}\text{u}$/water nanofluids velocity is influenced by boundary conditions in terms of entropy generation and heat transfer. M. Ghalambaz et al.^15^ considered thermo movement through a phase modification in a chamber heated downwards: using single or mixed nanoparticles as additives and their effects. Mehrayn et al.^16^ examinations of the mixed convective heat transfer between a square porous chamber containing an $\:{\text{A}\text{l}}_{2}{\text{O}}_{3}$-water nanofluids and a $\:\text{C}\text{u}-{\text{A}\text{l}}_{2}{\text{O}}_{3}$-water hybrid nanofluids. Using the Tiwari-Das nanofluids model, radiation and magnetic effects on mixed convection in an enclosure with $\:\text{T}\text{i}{\text{O}}_{2}$ nanoparticles were studied^17^. Reddy et al. investigated magneto nanofluids velocity in a square chamber infused with carbon nanotubes’ entropy generation and heat transfer^18^. Goswami et al.^19^ explored a deep enclosure containing multiple isothermal source-sink pairs and transient thermo gravitational velocity for MHD hybrid nanofluids.

Armaghani et al.^20^ considered a localized thermal source and sink in an $\:{\text{A}\text{l}}_{2}{\text{O}}_{3}-\text{C}\text{u}$ /water hybrid nanofluids in an L-shaped chamber using MHD mixed convection. Kumar et al.^48^ investigate the effects of heat generation and absorption in micro-rotating Casson’s nanofluid using a magnetohydrodynamics (MHD) model within porous media. Priyadashini and Sivaraj^21^ using numerical method, investigated the modeling of thermo-magnetic convection and entropy generation in a square enclosure infused with ferrofluid with the effect of a solid body. $\:{\text{A}\text{l}}_{2}{\text{O}}_{3}-{H}_{2}O$ nanofluids mixed convecting in an open, inclined cavity with a heat source^22^. An alumina-water nanofluids-infused square chamber involving a heat-conducting and heat-generating device has been studied numerically for mixed convection cooling^23^. Mansour et al.^24^ studied entropy generation and mixed convection velocity and heat transfer in a square chamber infused with the $\:\text{C}\text{u}-{\text{A}\text{l}}_{2}{\text{O}}_{3}$-Water hybrid nanofluids differentially heated and cooled by heat source and sink. Selimefendigil et al.^25^ used inclined magnetic force applied to top and bottom diagonal triangular domains in a lid-driven chamber infused with nanofluids produce mixed convection and entropy. Kaswan et al.^49^ investigate heat generation and absorption in nanofluid systems with activation energy. The research highlights how varying the radiation parameter impacts heat generation and absorption within the nanofluid. Agrawal and Kaswan^50^ investigate the heat transfer performance and flow characteristics of a hybrid nanofluid composed of water as the base fluid, between two parallel disks. Kumar et al.^52^ conducted a comprehensive analysis of steady-state momentum and heat transfer characteristics of hybrid nanofluids within a porous medium. Fazruddin et al.^26^ provided a numerical solution of the impact of different tilted positions of a thin fin on laminar viscous flow and mixed convection in a square enclosure. Vinodhini and Prasad^27^ numerically investigated the Buongiorno nanofluid flow in a rectangular cavity using the MAC method. Vinodhini and Prasad^28^ effectively determined the incompressible, unsteady, laminar, and natural convection that occurs in magnetic nanofluids in a two-dimensional square cavity. Sumithra and Sivaraj^29^ have discussed how chemically reactive MHD mixed convective nanofluids velocity occurs along with viscous dispersion and Ohmic heating, To describe the nano liquid, the Buongiorno mathematical model is used, and the influence of Brownian motion and thermophoresis are taken into account. Basak and Chamkha^30^ founded on the portrayal of thermo velocity using heat functions or heat lines, mixed convection of nanofluids in the existence of hot and cold side walls heating of the bottom horizontal wall with cold side walls have been explored. The Galerkin FEM has been used to solve velocity and temperature equations as well as post-process stream functions and heat functions. The investigation by Kumar et al.^51^ specifically addresses the impact of heat generation and absorption in nanofluid systems.

Rashidi et al.^31^ observed to examine the mixed convection in a square chamber containing an $\:{\text{A}\text{l}}_{2}{\text{O}}_{3}$/water nanofluids. When the bottom wall is displayed to heterogeneous heating, the right wall is regarded as cool, and the top wall and left wall are adiabatic, the velocity and heat transmission properties of the nanofluids in the enclosure are reported. There are nine alternative scenarios taken into consideration for the non-uniform heat flux, where the magnitude of the overall heat flux deployed to the enclosure is the same in each scenario but the profile varies. The common differentially heated square chamber problem is revisited to examine the effects of Non-Oberbeck-Boussinesq (NOB) on laminar mixed convection heat transfer to non-Newtonian power-law fluids. S. Rath conducted a numerical investigation of the improvement and diminished velocity and heat transfer characteristics achieved by the interaction of power-law rheology and NOB impacts^32^ using a finite volume approach. Utilizing nanofluids in a square chamber increases natural convective heat transfer while a magnetic force exists and there is uniform heat generation and absorption investigated by Teamah^33^. In a square chamber packed with a fluid-saturated permeable, the impacts of an angled magnetic force and heat generation on unsteady mixed convection have been explored numerically. The enclosure’s vertical right and left walls are maintained at a constant but varied temperature, while the horizontal top and horizontal bottom walls are adiabatically heated. A set of PDE’s and the related boundary conditions are used to numerically depict the physical issues. ADI (Alternative Direction Implicit) method is the basis of the finite-difference implicit scheme used by Revnic et al.^34^. The study conducted by Hashemi et al.^44^ focuses on a porous cavity that is shaped like an incinerator, specifically designed to analyse heat transfer. The studies by Dogonchi et al.^45^ and Seyyedi et al.^46^ examine natural convection in a cavity filled with nanofluid, focusing on the impact of the Hartmann number. Their research demonstrates that changing the Hartmann number affects convective flow patterns, with higher values reducing flow strength and heat transfer efficiency due to the effects of the magnetic field. Chamkha et al.^47^ examined the mixed convection heat transfer characteristics within a square-vented cavity in detail.

The absence of prior research on $\:Ag/GO-$Water hybrid nanofluids, as noted in the literature review, presents an intriguing opportunity for further investigation in the field of nanofluid science. Such a hybrid system, combining silver nanoparticles $\:\left(Ag\right)$, graphene oxide $\:\left(GO\right)$, and water, holds significant potential for a range of applications due to the unique properties inherent in each component. This established the originality of the present study, which is inspired by emerging applications of the nanofluid in electronic devices, (bio)sensors, biomedical applications, supercapacitors, membranes, energy storage devices, catalysts, and water purification. The square cavity employs the $\:Ag/GO$-Water hybrid nanofluids which narrates the laminar MHD mixed convection flow differentially heated cavity with complex boundary conditions in the influence of magnetic force. The enclosure is partially heated from the center of the horizontal bottom wall at temperature $\:{T}_{h}$ and cooled from the center of the top horizontal walls at temperature $\:{T}_{c}$, while other portions of the cavity horizontal walls are insulated. Also, vertical walls are maintained cool temperatures. The governing equations formulated for non-dimensional variables such as stream functions, isotherms functions, and heat generation have been solved by using the marker cell method with a finite difference scheme. computation has been carried out to analyse the impact of the Hartman number, Reynolds number, Richardson number, and heat generation location on streamline and isotherm profiles as well as the disparity of the mean Nusselt number. Hybrid nanofluids, like $\:Ag-GO$ nanofluids, significantly enhance thermal properties, leading to various engineering and industrial applications. In industrial heat exchangers and automotive radiators, they improve heat transfer efficiency and cooling performance. Solar energy systems, including solar collectors, benefit from better solar energy absorption and temperature management. They prevent overheating in electronics, maintaining optimal performance in CPUs, GPUs, and data centres. Biomedical uses include hyperthermia therapy for cancer treatment and targeted drug delivery systems. Hybrid nanofluids also improve geothermal energy extraction, wind turbine cooling, and nuclear reactor safety. In manufacturing, they enhance cooling and lubrication in machining, welding, and soldering processes, leading to better surface finishes and longer tool life. Overall, hybrid nanofluids boost thermal management and energy efficiency across various fields.

## Mathematical formulation

### Problem statement

**Fig. 1:**
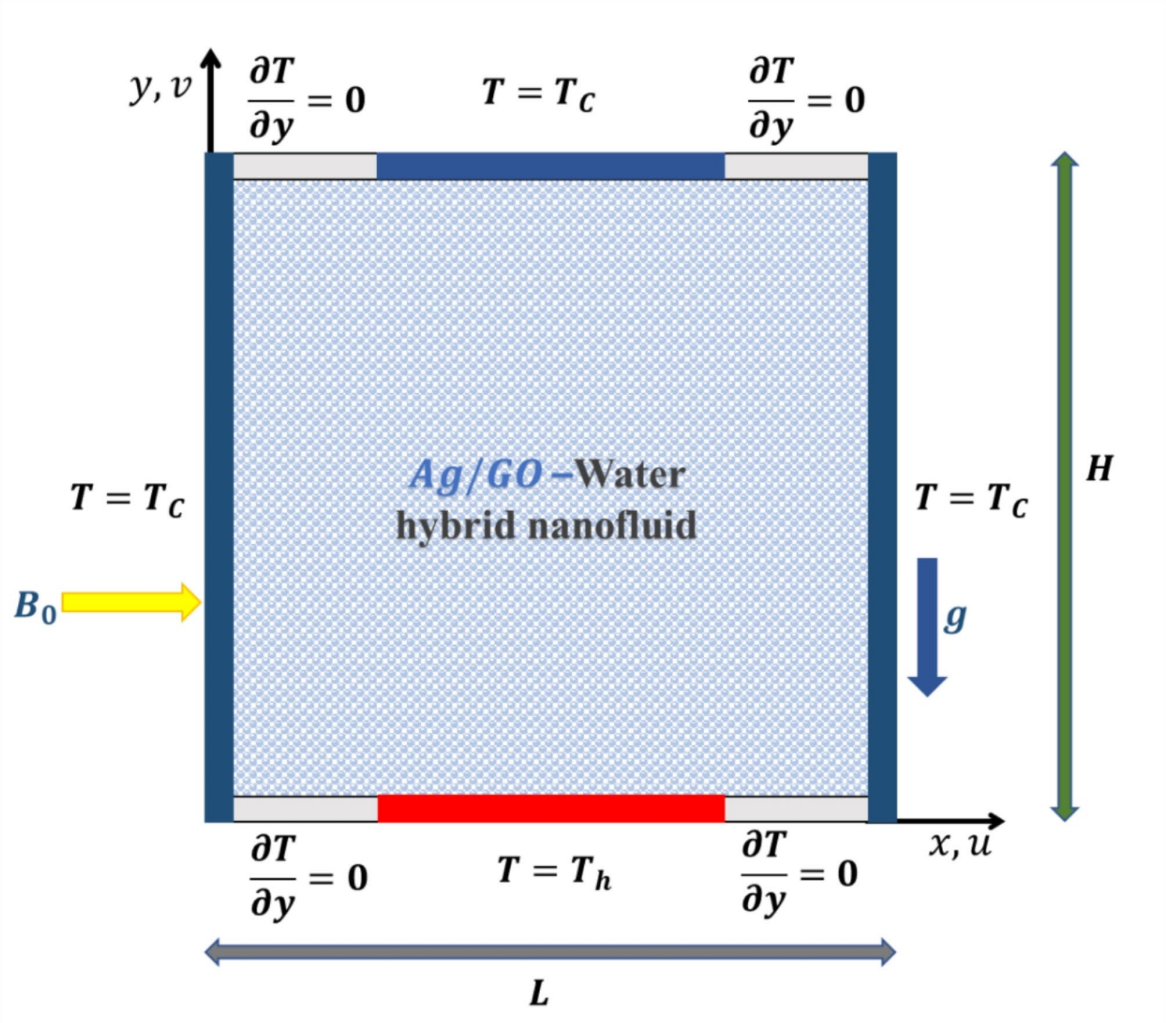
Flow geometry.

Figure 1 schematically describes the two-dimensional geometry that is studied in this manuscript. Mixed convective flow of an electrically conducting nanofluid in a square enclosure with a bottom and top wall of length $\:(L\:=\:H)$ nd a right and left wall of height $\:\left(H\right)\:$is considered. The right and left walls maintain a cooled temperature, while the middle portions of the top and bottom walls maintain an invariant temperature, $\:\left({T}_{c}\right)$ and $\:\left({T}_{h}\right)$, respectively, with the remaining portion of the cavity being adiabatic. The temperature T_h_ is higher than the temperature T_c_. The fluid flow in the square cavity is considered unsteady and laminar in nature. The thermophysical properties of the nanofluid considered $\:GO$, $\:Ag$ and $\:{H}_{2}O$ their properties are presented in Table 1. Two different nanofluids namely Ag (Silver) Graphene Oxide $\:\left(GO\right)$ nanofluids are investigated. The flow field is influenced by a transversely applied magnetic field. The impact of internal heat generation is taken into consideration. Magnetic nanofluid is considered dilute. The aqueous base fluid and the nanoparticles are in local thermal equilibrium. Hall current and magnetic induction effects are ignored.

### Dimensional equations:13,26,28,30,35

The following are the main conservation equations that define the hybrid-nanofluid region in the 2D cavity fluid domain:

1 $$\:\frac{\partial\:u}{\partial\:x}+\frac{\partial\:v}{\partial\:y}=0$$

2 $$\:\frac{\partial\:u}{\partial\:{t}^{\text{*}}}+u\frac{\partial\:u}{\partial\:x}+v\frac{\partial\:u}{\partial\:y}=-\frac{1}{{\rho\:}_{hnf}}\frac{\partial\:p}{\partial\:x}+\frac{{\mu\:}_{hnf}}{{\rho\:}_{hnf}}\left[\frac{{\partial\:}^{2}u}{{\partial\:x}^{2}}+\frac{{\partial\:}^{2}u}{{\partial\:y}^{2}}\right]\:$$

3 $$\:\frac{\partial\:v}{\partial\:{t}^{\text{*}}}+u\frac{\partial\:v}{\partial\:x}+v\frac{\partial\:v}{\partial\:y}=-\frac{1}{{\rho\:}_{hnf}}\frac{\partial\:p}{\partial\:y}+\frac{{\mu\:}_{hnf}}{{\rho\:}_{hnf}}\left[\frac{{\partial\:}^{2}v}{{\partial\:x}^{2}}+\frac{{\partial\:}^{2}v}{{\partial\:y}^{2}}\right]\:-\frac{{\sigma\:}_{hnf}{B}_{0}^{2}}{{\rho\:}_{hnf}}v+\frac{{\left(\rho\:\beta\:\right)}_{hnf}}{{\rho\:}_{hnf}}g(T-{T}_{c})$$

4 $$\:\frac{\partial\:T}{\partial\:{t}^{\text{*}}}+u\frac{\partial\:T}{\partial\:x}+v\frac{\partial\:T}{\partial\:y}={\alpha\:}_{hnf}\left[\frac{{\partial\:}^{2}T}{{\partial\:x}^{2}}+\frac{{\partial\:}^{2}T}{{\partial\:y}^{2}}\right]+\frac{{Q}_{0}}{{\left(\rho\:{C}_{p}\right)}_{hnf}}\left(T-{T}_{c}\right)$$

### Boundary conditions

Dimensional form of boundary conditions:^4–6,10,19^ &^24^.

**Table:** 

Left vertical wall $\:{t}^{*}>0,\:x=0,\:0\le\:y\le\:L,\:u=v=0\:\:\:\:\:\:\:\:\:\:\:\:\:\:\:\:\:\:T={T}_{c},\:\:\:\:for\:0\le\:y\le\:L\:\:\:\:$
Right vertical wall $\:{t}^{*}>0,\:x=L,\:0\le\:y\le\:L,\:u=v=0\:\:\:\:\:\:\:\:\:\:\:\:\:\:\:\:\:\:T={T}_{c},\:\:\:\:for\:0\le\:y\le\:L$
Bottom horizontal wall $$\:{\:t}^{*}>0,\:y=0,\:0\le\:x\le\:L,u=v=0$$$$\:\begin{array}{c}\frac{\partial\:T}{\partial\:y}=0,\:\:\:\:\:for\:\:\:\:\:0\le\:x\le\:\frac{L}{4}\\\:T={T}_{h},\:\:\:\:for\:\:\:\:\:\frac{L}{4}\le\:x\le\:\frac{3 L}{4}\\\:\frac{\partial\:T}{\partial\:y}=0,\:\:\:\:\:for\:\:\:\:\:\frac{3 L}{4}\le\:x\le\:L\end{array}$$
Top horizontal wall $$\:{t}^{*}>0,\:y=L,\:0\le\:x\le\:L,\:u=v=0\:\:\:\:\:\:\:\:\:\:\:\:\:\:\begin{array}{c}\frac{\partial\:T}{\partial\:y}=0,\:\:\:\:\:for\:\:\:\:\:0\le\:x\le\:\frac{L}{4}\\\:T={T}_{c},\:\:\:\:for\:\:\:\:\:\frac{L}{4}\le\:x\le\:\frac{3 L}{4}\\\:\frac{\partial\:T}{\partial\:y}=0,\:\:\:\:\:for\:\:\:\:\:\frac{3 L}{4}\le\:x\le\:L\end{array}$$

#### Thermophysical properties of hybrid nanofluids

T^24,36,39^ &^41^.

The following formulas will be used to determine the effective properties of the nanofluid implemented in these equations:

Hybrid nanofluids density:

5 $$\:{\rho\:}_{hnf}={(1-\varphi\:)\rho\:}_{bf}+{\varphi\:}_{GO}{\rho\:}_{GO}+{\varphi\:}_{Ag}{\rho\:}_{Ag}$$

Hybrid nanofluids heat capacitance:

6 $$\:{\left(\rho\:{C}_{p}\right)}_{hnf}={\left(1-\varphi\:\right)\left(\rho\:{C}_{p}\right)}_{bf}+{\varphi\:}_{GO}{\left(\rho\:{C}_{p}\right)}_{GO}+{\varphi\:}_{Ag}{\left(\rho\:{C}_{p}\right)}_{Ag}$$

Hybrid nanofluids thermal expansion coefficient:

7 $$\:{\left(\rho\:\beta\:\right)}_{hnf}=\left(1-\varphi\:\right){\left(\rho\:\beta\:\right)}_{bf}+{\varphi\:}_{GO}{\left(\rho\:\beta\:\right)}_{GO}+{\varphi\:}_{Ag}{\left(\rho\:\beta\:\right)}_{Ag}$$

Hybrid nanofluids thermal diffusivity:

8 $$\:{\alpha\:}_{hnf}=\frac{{k}_{hnf}}{{\left(\rho\:{C}_{p}\right)}_{hnf}}$$

Hybrid nanofluids thermal conductivity (Maxwell model):

9 $$\:\frac{{k}_{hnf}}{{k}_{bf}}=\left(\frac{\left({\varphi\:}_{GO}{k}_{GO}+{\varphi\:}_{Ag}{k}_{Ag}\right)}{\varphi\:}+2{k}_{bf}+2\left({\varphi\:}_{GO}{k}_{GO}+\\{\varphi\:}_{Ag}{k}_{Ag}\right)-2\varphi\:{k}_{bf}\right)\times\:{\left(\frac{\left({\varphi\:}_{GO}{k}_{GO}+{\varphi\:}_{Ag}{k}_{Ag}\right)}{\varphi\:}+\\2{k}_{bf}-\left({\phi\:}_{GO}{k}_{GO}+{\phi\:}_{Ag}{k}_{Ag}\right)+\phi\:{k}_{bf}\right)^{-1}}$$

Hybrid nanofluids dynamic viscosity (Brinkman model):

10 $$\:{\mu\:}_{hnf}=\frac{{\mu\:}_{bf}}{{\left(1-({\varphi\:}_{GO}+{\phi\:}_{Ag})\right)}^{2.5}}$$

Hybrid nanofluid’s electrical conductivity (Maxwell model):

11 $$\:\frac{{\sigma\:}_{hnf}}{{\sigma\:}_{bf}}=1+\frac{3\left(\frac{\left({\varphi\:}_{GO}{\sigma\:}_{GO}+{\varphi\:}_{Ag}{\sigma\:}_{Ag}\right)}{{\sigma\:}_{bf}}-\left({\varphi\:}_{GO}+{\phi\:}_{Ag}\right)\right)}{\left(\frac{\left({\varphi\:}_{GO}{\sigma\:}_{GO}+{\varphi\:}_{Ag}{\sigma\:}_{Ag}\right)}{\varphi\:{\sigma\:}_{bf}}+2\right)-\left(\frac{\left({\varphi\:}_{GO}{\sigma\:}_{GO}+{\varphi\:}_{Ag}{\sigma\:}_{Ag}\right)}{{\sigma\:}_{bf}}-\left({\varphi\:}_{GO}+{\phi\:}_{Ag}\right)\right)}\:\:$$

**Table 1:** Thermophysical properties of $GO$, $\:Ag$ and $\:{H}_{2}O$^36,38,39^ &^41^.

Property	H_2_O	*Ag* (Silver)	Graphene Oxide (GO)
$\:\rho\:(\text{k}\text{g}{m}^{-3}$)	998.2	10,500	1800
$\:{C}_{p}(\text{J}{kg}^{-1}{K}^{-1})$	4179	235	717
k(W$\:{m}^{-1}{k}^{-1}$)	0.613	429	5000
$\:\beta\:({k}^{-1}$)	21 × 10^−5^	1.89 × 10^−5^	2.84 × 10^−4^
$\:\sigma\:\left({{\Omega\:}}^{-1}{m}^{-1}\right)$	0.05	63 × 10^6^	6.30 × 10^7^
$\:\mu\:\left(Pa.\:s\right)$	8.9 × 10^−4^	–	–
Pr	6.2	–	–

The Eq. (1) through (4) are converted into the non-dimensional form by implementing the dimensionless quantities listed below:

$$\:X=\frac{x}{L}$$; $$\:Y=\frac{y}{L}$$; $$\:t=\frac{{t}^{*}{U}_{0}}{L}$$; $$\:U=\frac{u}{{U}_{0}}$$; $$\:V=\frac{v}{{U}_{0}}$$; $$\:P=\frac{p}{{\rho\:}_{hnf}{U}_{0}^{2}}$$; $$\:\theta\:=\frac{T-{T}_{c}}{{T}_{h}-{T}_{c}}$$

**Non-dimensional form of Governing equations**:

12 $$\:\frac{\partial\:U}{\partial\:X}+\frac{\partial\:V}{\partial\:Y}=0$$

13 $$\:\frac{\partial\:U}{\partial\:t}+U\frac{\partial\:U}{\partial\:X}+V\frac{\partial\:U}{\partial\:Y}=-\frac{\partial\:P}{\partial\:X}+\left(\frac{{\nu\:}_{hnf}}{{\nu\:}_{bf}}\right)\frac{1}{Re}\left[\frac{{\partial\:}^{2}U}{\partial\:{X}^{2}}+\frac{{\partial\:}^{2}U}{\partial\:{Y}^{2}}\right]$$

14 $$\:\frac{\partial\:V}{\partial\:t}+U\frac{\partial\:V}{\partial\:X}+V\frac{\partial\:V}{\partial\:Y}=-\frac{\partial\:P}{\partial\:Y}+\left(\frac{{\nu\:}_{hnf}}{{\nu\:}_{bf}}\right)\frac{1}{Re}\left[\frac{{\partial\:}^{2}V}{\partial\:{X}^{2}}+\frac{{\partial\:}^{2}V}{\partial\:{Y}^{2}}\right]\:-\:\left(\frac{{\rho\:}_{bf}}{{\rho\:}_{hnf}}\right)\left(\frac{{\sigma\:}_{hnf}}{{\sigma\:}_{bf}}\right)\frac{H{a}^{2}}{Re}\text{V}+\frac{{\left(\rho\:\beta\:\right)}_{hnf}}{{\rho\:}_{hnf}{\beta\:}_{bf}}\:Ri\:\theta\:$$

15 $$\:\frac{\partial\:\theta\:}{\partial\:t}+U\frac{\partial\:\theta\:}{\partial\:X}+V\frac{\partial\:\theta\:}{\partial\:Y}=\frac{{\alpha\:}_{hnf}}{{\alpha\:}_{bf}}\frac{1}{{Pr}Re}\left[\frac{{\partial\:}^{2}\theta\:}{{\partial\:X}^{2}}+\frac{{\partial\:}^{2}\theta\:}{{\partial\:Y}^{2}}\right]+\frac{{\alpha\:}_{hnf}}{{\alpha\:}_{bf}}\frac{1}{Re\:Pr}Q\theta\:$$

The corresponding dimensionless specifications are provided in Table 2.

**Table 2:** Pertinent parameters.

$\:Pr=\frac{{\nu\:}_{bf}}{{\alpha\:}_{bf}}$	Prandtl Number
$\:Ha={B}_{0}L\sqrt{\frac{{\sigma\:}_{bf}}{{\mu\:}_{bf}}}$	Hartmann Number
$\:Gr=\frac{g{\beta\:}_{bf}\left({T}_{h}-{T}_{c}\right){L}^{3}}{{{\nu\:}_{bf}\:}^{2}}$	Grashof Number
$\:Re=\frac{{U}_{0}L}{{\nu\:}_{bf}}$	Reynolds Number
$\:Ri=\frac{Gr}{{Re}^{2}}$	Richardson Number
$\:Q=\frac{{Q}_{0}\:{L}^{2}}{{\left({\rho\:C}_{p}\right)}_{bf}{\alpha\:}_{bf}}$	Heat generation/absorption Coefficient

### Non-dimensional form

#### Initial conditions

$$\:t\le\:0,\:\:0\le\:X\le\:1,\:\:0\le\:Y\le\:1,\:\:U=V=0,\:\:\theta\:=0$$

#### Boundary conditions

Table 3 presents the non-dimensional boundary conditions applied to the cavity walls.

**Table 3:** Non-dimensional boundary conditions.

Left vertical wall$\:t>0,X=0,\:0\le\:Y\le\:1,U=V=0$,$\:\theta\:=0\:\:for\:\:\:\:\:0\le\:Y\le\:1$
Right vertical wall$\:\:\:\:t>0,X=1,\:0\le\:Y\le\:1,\:U=V=0,\:\:\:\:\:\:\:\:\:\theta\:=0\:\:for\:\:\:\:\:0\le\:Y\le\:1$
Bottom vertical wall$\:\:t>0,\:\:Y=0,\:0\le\:X\le\:1,U=V=0$,$$\:\begin{array}{c}\frac{\partial\:\theta\:}{\partial\:Y}=0,\:\:\:\:\:for\:\:\:\:\:0\le\:X\le\:\frac{1}{4}\\\:\:\theta\:=1,\:\:\:\:\:for\:\:\:\:\:\frac{1}{4}\le\:X\le\:\frac{3}{4}\\\:\frac{\partial\:\theta\:}{\partial\:Y}=0,\:\:\:\:\:for\:\:\:\:\frac{3}{4}\le\:X\le\:1\end{array}$$
Top vertical wall$$\:t>0,\:\:Y=1,\:\:0\le\:X\le\:1,U=V=0,\:\:\:\:\:\begin{array}{c}\frac{\partial\:\theta\:}{\partial\:Y}=0,\:\:\:\:\:for\:\:\:\:\:0\le\:X\le\:\frac{1}{4}\\\:\:\theta\:=0,\:\:\:\:\:for\:\:\:\:\:\frac{1}{4}\le\:X\le\:\frac{3}{4}\\\:\frac{\partial\:\theta\:}{\partial\:Y}=0,\:\:\:\:\:for\:\:\:\:\frac{3}{4}\le\:X\le\:1\end{array}$$

### Nusselt number

**Local Nusselt number**:

16 $$\:Nu=-\frac{{k}_{hnf}}{{k}_{bf}}{\left(\frac{\partial\:\theta\:}{\partial\:Y}\right)}_{Y=0}$$

**Average Nusselt number**:

17 $$\:{Nu}_{avg}=\underset{0}{\overset{1}{\int\:}}Nu\:dX$$

## Method of solution

### MAC formulation

**Fig. 2:**
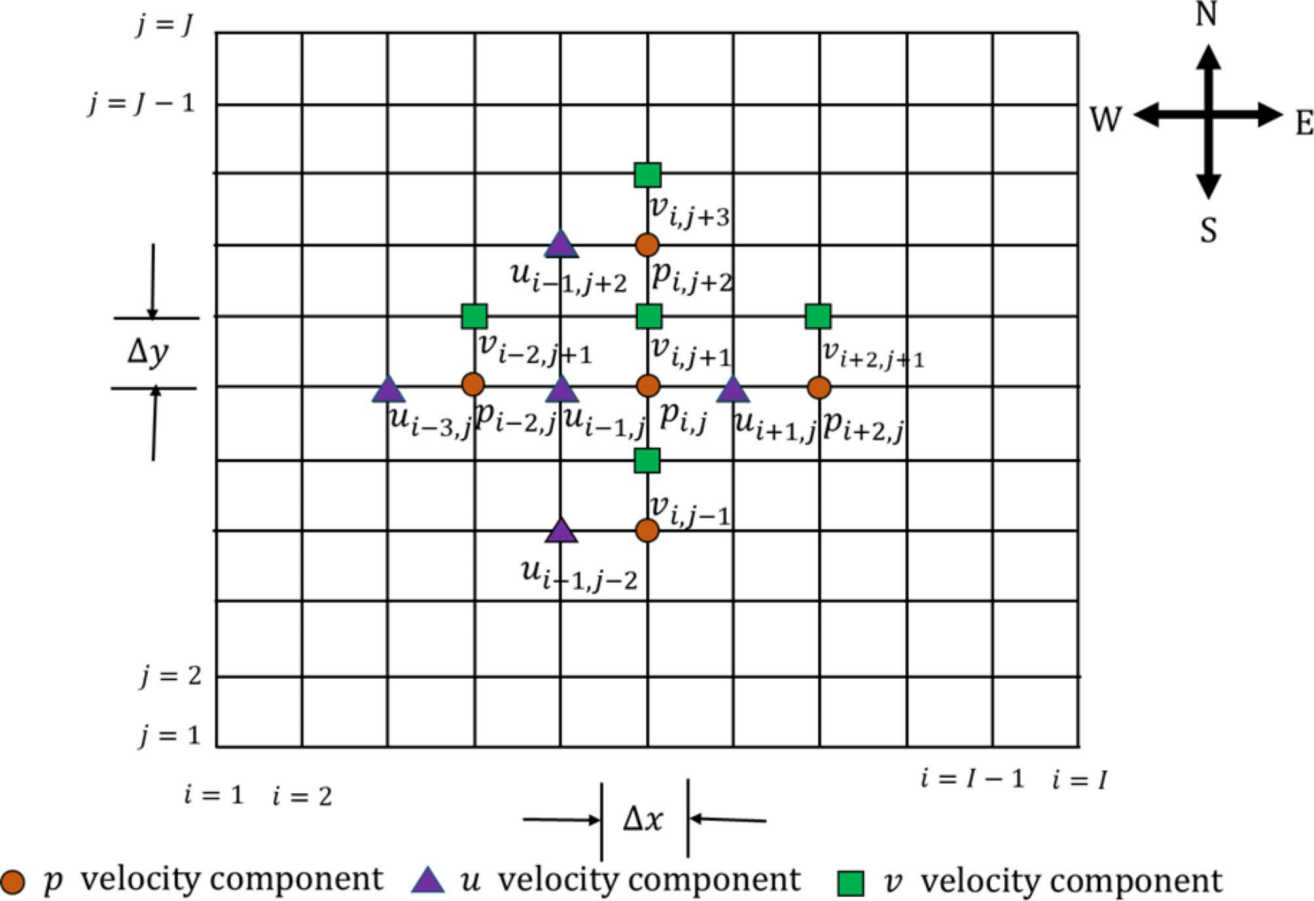
The staggered grid system used for MAC method.

The area in which estimations are to be executed is split into a set of tiny cells having edges that indicate $\:\varDelta\:x,\varDelta\:y.\:$Regarding this set of computational cells, Pressure and temperature are specified at the center of the cells, while components of velocity are situated at the center of the cell faces. Cells are defined with an index $\:(i,j),$ Also, $\:{p}_{i,j}$ is the pressure at the center of the cell $\:(i,j),$ while $\:{u}_{i,j}$ is the $\:x-$direction velocity at the center of the face between cells open $\:(i,j)$ and $\:(i+1,j)$ and so on. According to the staggered grid structures, the velocities are not specified at the nodes, but once needed; they can be located through interpolation. Figure 2 illustrates the staggered grid system used for the MAC method.

We can say, for example, when using uniform grids

18 $$\:{u}_{i-\frac{1}{2}j}=\frac{1}{2}\left[{u}_{i-1,\:\:j}+{u}_{i,j}\right]\:\:$$

A weighted average of second upwind and space-centered schemes are utilized to discretize convective terms. (Hirt et al.,1975).

By using a central difference scheme, diffusive terms are discretized.

Suppose the non-dimensional $\:U-$ velocity equation is:

19 $$\:\frac{\partial\:u}{\partial\:t}+\frac{\partial\:\left({u}^{2}\right)}{\partial\:x}+\frac{\partial\:\left(uv\right)}{\partial\:y}=-\frac{\partial\:p}{\partial\:x}+\frac{1}{Re}\left[\frac{{\partial\:}^{2}u}{\partial\:{x}^{2}}+\frac{{\partial\:}^{2}u}{\partial\:{y}^{2}}\right]$$

20 $$\:\frac{\partial\:\left({u}^{2}\right)}{\partial\:x}=\frac{1}{4\varDelta\:x}\left[\begin{array}{c}\left({u}_{i,j}+{u}_{i+1,j}\right)\left({u}_{i,j}+{u}_{i+1,j}\right)+\alpha\:\left|\left({u}_{i,j}+{u}_{i+1,j}\right)\right|\left({u}_{i,j}-{u}_{i+1,j}\right)\\\:-\left({u}_{i-1,j}+{u}_{i,j}\right)\left({u}_{i-1,j}+{u}_{i,j}\right)-\alpha\:\left|\left({u}_{i-1,j}+{u}_{i,j}\right)\right|\left({u}_{i-1,j}-{u}_{i,j}\right)\end{array}\right]=DUUDX$$

21 $$\:\frac{\partial\:\left(uv\right)}{\partial\:y}=\frac{1}{4\varDelta\:y}\left[\begin{array}{c}\left({v}_{i,j}+{v}_{i+1,j}\right)\left({u}_{i,j}+{u}_{i,j+1}\right)+\alpha\:\left|\left({v}_{i,j}+{v}_{i+1,j}\right)\right|\left({u}_{i,j}-{u}_{i,j+1}\right)\\\:-\left({v}_{i,j-1}+{v}_{i+1,j-1}\right)\left({u}_{i,j-1}+{u}_{i,j}\right)-\alpha\:\left|\left({v}_{i,j-1}+{v}_{i+1,j-1}\right)\right|\left({u}_{i,j-1}-{u}_{i,j}\right)\end{array}\right]=DUVDY$$

22 $$\:\frac{\partial\:p}{\partial\:x}=\frac{{p}_{i+1,j}-{p}_{i,j}}{\varDelta\:x}\equiv\:DPDX$$

23 $$\:\frac{{\partial\:}^{2}u}{\partial\:{x}^{2}}=\frac{{u}_{i+1,j}-2{u}_{i,j}+{u}_{i-1,j}}{{\left(\varDelta\:x\right)}^{2}}\equiv\:D2UDX2$$

24 $$\:\frac{{\partial\:}^{2}u}{\partial\:{y}^{2}}=\frac{{u}_{i,j+1}-2{u}_{i,j}+{u}_{i,j-1}}{{\left(\varDelta\:y\right)}^{2}}\equiv\:D2UDY2$$

With $\:\alpha\:\to\:1$ Scheme $\:\to\:$ Second upwind

$\:\alpha\:\to\:0$ Scheme $\:\to\:$ Second centered

It is thought that we obtain a converged result at the level $\:t={n}^{th}$. Then, for the subsequent time step

25 $$\:\frac{{\stackrel{\sim}{u}}_{i,j}^{n+1}-{u}_{i,j}^{n}}{\varDelta\:t}={\left[SDISCU\right]}_{i,j}^{n}$$

Where.

$\:{\left[SDISCU\right]}_{i,j}^{n}$=*spatial discretization of the convective and diffusive terms*.

The quantity $\:{\stackrel{\sim}{u}}_{i,j}^{n+1}$ is now calculated explicitly from the discretized form as

26 $$\:{\stackrel{\sim}{u}}_{i,j}^{n+1}={u}_{i,j}^{n}+\varDelta\:t{\left[SDISCU\right]}_{i,j}^{n}$$

Where

27 $$\:{\left[SDISCU\right]}_{i,j}^{n}=\left(-DUUDX-DUVDY\right)-DPDX+\left(1/\text{R}\text{e}\text{\hspace{0.33em}}\right)\left(D2UDX2+D2UDY2\right)$$

Similarly, We evaluate

28 $$\:{\stackrel{\sim}{v}}_{i,j}^{n+1}={v}_{i,j}^{n}+\varDelta\:t{\left[SDISCV\right]}_{i,j}^{n}$$

- The explicitly advanced velocities possibly not consistently generate a fluid field over a zero-mass divergence in every cell. The pressure distribution is obviously incorrect at this point, as indicated by this.
- The correction of each cell’s pressure ensures that there does not exist net mass flow either inside or outside of the cell.
- In the MAC method, the corrected pressure was determined by solving a Poisson pressure equation.
- i.e. The iterative correction process to yield a divergence-free velocity field.

The computational technique of this iterative pressure-velocity correction process is given as below:

The connection between velocity at the previous time step and the explicitly advanced velocity component exists to be displayed as

29 $$\:{\stackrel{\sim}{u}}_{i,j}^{n+1}={u}_{i,j}^{n}+\varDelta\:t\frac{\left[{p}_{i,j}^{n}-{p}_{i+1,j}^{n}\right]}{\delta\:x}+\varDelta\:t{\left[CONDIFU\right]}_{i,j}^{n}$$

Where $$\:{\left[CONDIFU\right]}_{i,j}^{n}={\left[SDISCU\right]}_{i,j}^{n}+{\left[DPDX\right]}_{i,j}^{n}$$

On the contrary, the correct pressure component (also unknown) will be related to the corrected velocity component in the manner illustrated below:

30 $$\:{u}_{i,j}^{n+1}={u}_{i,j}^{n}+\varDelta\:t\frac{\left[{p}_{i,j}^{n+1}-{p}_{i+1,j}^{n+1}\right]}{\varDelta\:x}+\varDelta\:t{\left[CONDIFU\right]}_{i,j}^{n}$$

From Eqs. (29) and (30)

31 $$\:{u}_{i,j}^{n+1}-{\stackrel{\sim}{u}}_{i,j}^{n+1}=\frac{\varDelta\:t\left[{p}_{i,j}^{{\prime\:}}-{p}_{i+1,j}^{{\prime\:}}\right]}{\varDelta\:x}$$

When the pressure correction may be described as

32 $$\:{p}_{i,j}^{{\prime\:}}={p}_{i,j}^{n+1}-{p}_{i+1,j}^{n}$$

Neither the pressure corrections nor $\:{u}_{i,j}^{n+1}$ are known explicitly at this stage. Consequently, the Calculations are carried out in an iterative loop.

We can formulate the following:

**Table:** 

Corrected	estimated	Correction
$\:{u}_{i,j}^{n+1}$	$\:\to\:{\stackrel{\sim}{u}}_{i,j}^{n+1}$	$\:+\frac{\varDelta\:t\left[{p}_{i,j}^{{\prime\:}}-{p}_{i+1,j}^{{\prime\:}}\right]}{\varDelta\:x}$
$\:{u}_{i-1,j}^{n+1}$	$\:{\to\:\stackrel{\sim}{u}}_{i-1,j}^{n+1}$	$\:\:\:\:\:\:\:\:-\frac{\varDelta\:t\left[{p}_{i,j}^{{\prime\:}}-{p}_{i-1,j}^{{\prime\:}}\right]}{\varDelta\:x}\:$
$\:{v}_{i,j}^{n+1}$	$\:{\to\:\stackrel{\sim}{v}}_{i,j}^{n+1}$	$\:+\frac{\varDelta\:t\left[{p}_{i,j}^{{\prime\:}}-{p}_{i,j+1}^{{\prime\:}}\right]}{\varDelta\:y}$
$\:{v}_{i,j-1}^{n+1}$	$\:{\to\:\stackrel{\sim}{v}}_{i,j-1}^{n+1}$	$\:-\frac{\varDelta\:t\left[{p}_{i,j}^{{\prime\:}}-{p}_{i,j-1}^{{\prime\:}}\right]}{\varDelta\:y}$

The continuity equation is employed to calculate the correction. When the previously mentioned interactions are plugged into the continuity equation,

33 $$\:\frac{\partial\:u}{\partial\:x}+\frac{\partial\:v}{\partial\:y}=0$$

$$\:\left[\frac{{u}_{i,j}^{n+1}-{u}_{i-1,j}^{n+1}}{\varDelta\:x}+\frac{{v}_{i,j}^{n+1}-{v}_{i,j-1}^{n+1}}{\varDelta\:y}\right]\:\:\:\:=\left[\frac{{\stackrel{\sim}{u}}_{i,j}^{n+1}-{\stackrel{\sim}{u}}_{i-1,j}^{n+1}}{\varDelta\:x}+\frac{{\stackrel{\sim}{v}}_{i,j}^{n+1}-{\stackrel{\sim}{v}}_{i,j-1}^{n+1}}{\varDelta\:y}\right]\:\:\:\:\:-\varDelta\:t\left[\frac{{{p}^{{\prime\:}}}_{i+1,j}-2{{p}^{{\prime\:}}}_{i,j}+{{p}^{{\prime\:}}}_{i-1,j}}{{\left(\varDelta\:x\right)}^{2}}+\frac{{{p}^{{\prime\:}}}_{i,j+1}-2{{p}^{{\prime\:}}}_{i,j}+{{p}^{{\prime\:}}}_{i,j-1}}{{\left(\varDelta\:y\right)}^{2}}\right]$$

(or)

34 $$\left[ {\frac{{u_{{i,j}}^{{n+1}} - u_{{i - 1,j}}^{{n+1}}}}{{\Delta x}}+\frac{{v_{{i,j}}^{{n+1}} - v_{{i,j - 1}}^{{n+1}}}}{{\Delta y}}} \right]=\left[ {\frac{{\tilde {u}_{{i,j}}^{{n+1}} - \tilde {u}_{{i - 1,j}}^{{n+1}}}}{{\Delta x}}+\frac{{\tilde {v}_{{i,j}}^{{n+1}} - \tilde {v}_{{i,j - 1}}^{{n+1}}}}{{\Delta y}}} \right]+\frac{{2\Delta t({{p^{\prime}}_{i,j}})}}{{\Delta {x^2}}}+\frac{{2\Delta t({{p^{\prime}}_{i,j}})}}{{\Delta {y^2}}}$$

The above expression is derived under the expectation that the pressure corrections through cells nearby are zero. The divergence of the velocity field in a finite difference form is the first term enclosed in square brackets.

It is given a symbol $\:{\left(Div\right)}_{i,j}$ Returning to the algebraic calculations, we can write

35 $$0={(Div)_{i,j}}+{p^{\prime}_{i,j}}\left[ {2\Delta t\left( {\frac{1}{{\Delta {x^2}}}+\frac{1}{{\Delta {y^2}}}} \right)} \right]$$

Or.

36 $${p^{\prime}_{i,j}}=\frac{{ - {{(Div)}_{i,j}}}}{{\left[ {2\Delta t\left( {\frac{1}{{\Delta {x^2}}}+\frac{1}{{\Delta {y^2}}}} \right)} \right]}}$$

The pressure correction equation adjusts in the following order to accelerate the calculation:

37 $${p^{\prime}_{i,j}}=\frac{{ - {\omega _o}{{(Div)}_{i,j}}}}{{\left[ {2\Delta t\left( {\frac{1}{{\Delta {x^2}}}+\frac{1}{{\Delta {y^2}}}} \right)} \right]}}$$

Where $\:{\omega\:}_{0}$ defined the over-relaxation factor. By using numerical experimentation, among should be determined the value of $\:{\omega\:}_{0}$ takes the shortest time to converge.

After calculating $\:{p}_{i,j}^{{\prime\:}}\to\:$ the pressure cell is $\:(i,j)$ adjusted as

38 $$p_{{i,j}}^{{n+1}} \to p_{{i,j}}^{n}+{p^{\prime}_{i,j}}$$

At present, the velocity and pressure elements for every cell are adjusted for an iterative approach, throughout a manner that of end pressure gradient, the velocity divergence in every cell disappears.

The procedure proceeds until a divergence-free momentum field is connected to a suggested maximum range.

A discretization approach for the $\:U$- velocity equation for the subsequent next time step $\:\left({U}^{n+1}\right)$ is displayed next:

39 $$\:{U}^{n+1}={U}^{n}+dt\left\{\begin{array}{c}-\left(U\frac{\partial\:U}{\partial\:X}+V\frac{\partial\:U}{\partial\:Y}\right)+\left(\frac{{\nu\:}_{hnf}}{{\nu\:}_{bf}}\right)\frac{1}{Re}\left(\frac{{\partial\:}^{2}U}{\partial\:{X}^{2}}+\frac{{\partial\:}^{2}U}{\partial\:{Y}^{2}}\right)\\\:\:\end{array}\right\}$$

A discretization approach for the $\:V$- velocity equation for the subsequent time step $\:\:{V}^{n+1}$requires a small modification through the addition of yet another term, and it involves the following form:

40 $$\:{V}^{n+1}={V}^{n}+dt\left[\begin{array}{c}-\left(U\frac{\partial\:V}{\partial\:X}+V\frac{\partial\:V}{\partial\:Y}\right)+\left(\frac{{\nu\:}_{hnf}}{{\nu\:}_{bf}}\right)\frac{1}{Re}\left[\frac{{\partial\:}^{2}V}{\partial\:{X}^{2}}+\frac{{\partial\:}^{2}V}{\partial\:{Y}^{2}}\right]\:-\:\left(\frac{{\rho\:}_{bf}}{{\rho\:}_{hnf}}\right)\left(\frac{{\sigma\:}_{hnf}}{{\sigma\:}_{bf}}\right)\frac{H{a}^{2}}{Re}V+\frac{{\left(\rho\:\beta\:\right)}_{hnf}}{{\rho\:}_{hnf}{\beta\:}_{bf}}\:Ri\:\theta\:\\\:\:\end{array}\right]$$

While using the previous equation, the energy $\:\left(\theta\:\right)$ is co-located to reflect the staggered grid. After projecting $\:{U}^{n+1}$ and $\:{V}^{n+1}$, $\:U\:$and $\:V$ can be estimated using the Poisson-pressure Eq. 

41 $$\:\frac{{\nabla\:}^{2}U}{dt}={\nabla\:}^{2}p$$

The energy equation will be discretized for the subsequent time step $\:{(\theta\:}^{n+1})$ is obtained by as below:

42 $$\:{\theta\:}^{n+1}={\theta\:}^{n}+\varDelta\:t\left(-\left(U\frac{\partial\:\theta\:}{\partial\:X}+V\frac{\partial\:\theta\:}{\partial\:Y}\right)+\frac{{\alpha\:}_{hnf}}{{\alpha\:}_{bf}}\frac{1}{{Pr}Re}\left[\frac{{\partial\:}^{2}\theta\:}{{\partial\:X}^{2}}+\frac{{\partial\:}^{2}\theta\:}{{\partial\:Y}^{2}}\right]+\frac{{\alpha\:}_{hnf}}{{\alpha\:}_{bf}}\frac{1}{Re\:Pr}Q\theta\:\:\right)$$

### Validation and evaluation of grid independence

**Fig. 3:**
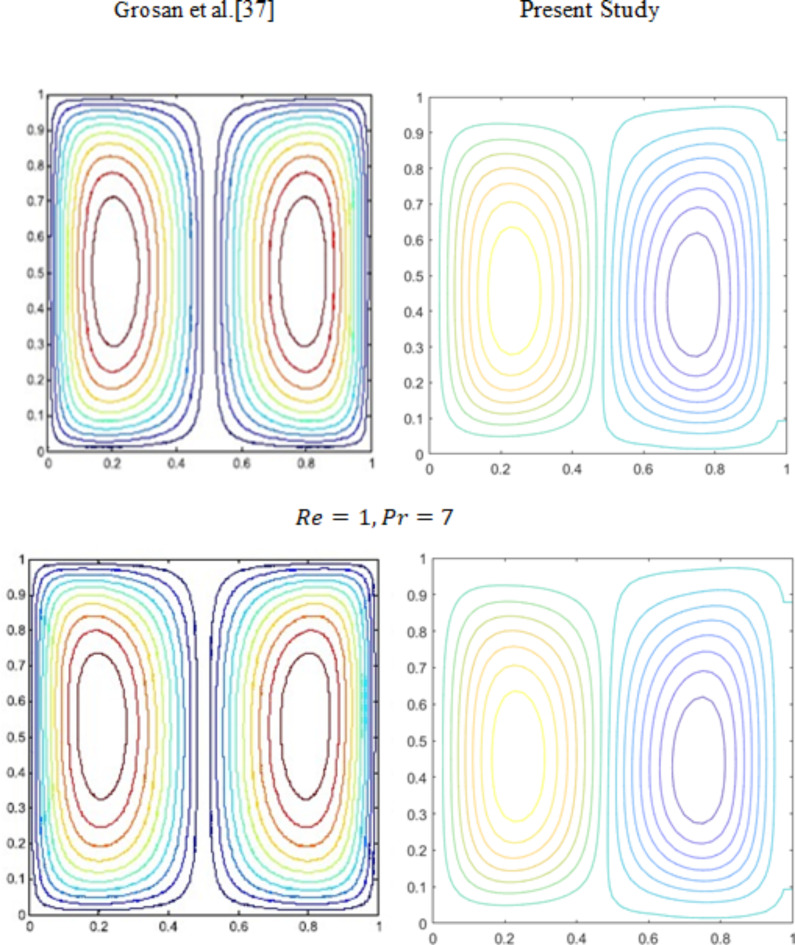
Comparison of streamlines contour plots with $\:\:Re=1$, and $\:Pr=7$.

Figure 3 illustrates the comparison between streamlines with different values of heat generation/absorption parameter number compared with Grosan et al.^37^. These essentially reprise Grosan et al. solutions^37^. The overall comparison result shows a high level of agreement. The present study is validated using the MAC algorithm code, which indicates that the study is more appropriate with the existing results. Table 4 illustrates the comparison of the average Nusselt number with values of the Hartmann number and volume fraction values compared with Ghasemi et al.^42^ and Rajarathinam et al.^43^.

**Table 4:** Comparison table for average Nusselt number for different values of $\:Ha$ and $\:\varphi\:$.

$\:Ha$	$\:\varphi\:$	$\:\stackrel{-}{Nu}$		
		Ghasemi et al. [42]	Rajarathinam et al. [43]	Present Work
0	0.0	4.738	4.754	4.758
	0.02	4.820	4.833	4.837
	0.04	4.896	4.909	4.911
45	0.0	2.365	2.392	2.394
	0.02	2.342	2.365	2.370
	0.04	2.317	2.339	2.342

**Table 5:** Grid-independent test for average nusselt number ($\:N{u}_{Avg})$.

Grid size	$\:N{u}_{Avg}$
40 × 40	1.4263
50 × 50	3.0266
80 × 80	4.0050
100 × 100	4.3846

Table 5 illustrates the manner in which the highest possible value and mean Nusselt number of stream functions improve as the total number of nodes grows. The findings of an independent grid study suggested that a grid over elements numbered $\:100$ through $\:100$ would be appropriate for displaying the outcomes. The influences used thought of in this investigation are $\:Pr=6.2,Q=-10,\:Re=10,\:Ri=10\:\text{a}\text{n}\text{d}\:Ha=10.$

## Interpretation of results

This part discusses the outcomes of simulating the current problem of the $\:Ag/GO-$water hybrid nanofluids flow pattern on the mixed convective heat transfer properties. The effects of the non-dimensional parameters that are kept constant, which include the Reynolds number $\:(0\le\:Re\le\:30)$, Richardson number $\:(0.1\le\:Ri\le\:10),$ Hartmann number $\:(0\le\:Ha\le\:50)$ and heat generation/absorption $\:(-10\le\:Q\le\:10).$

### Hartmann number effects

**Fig. 4:**
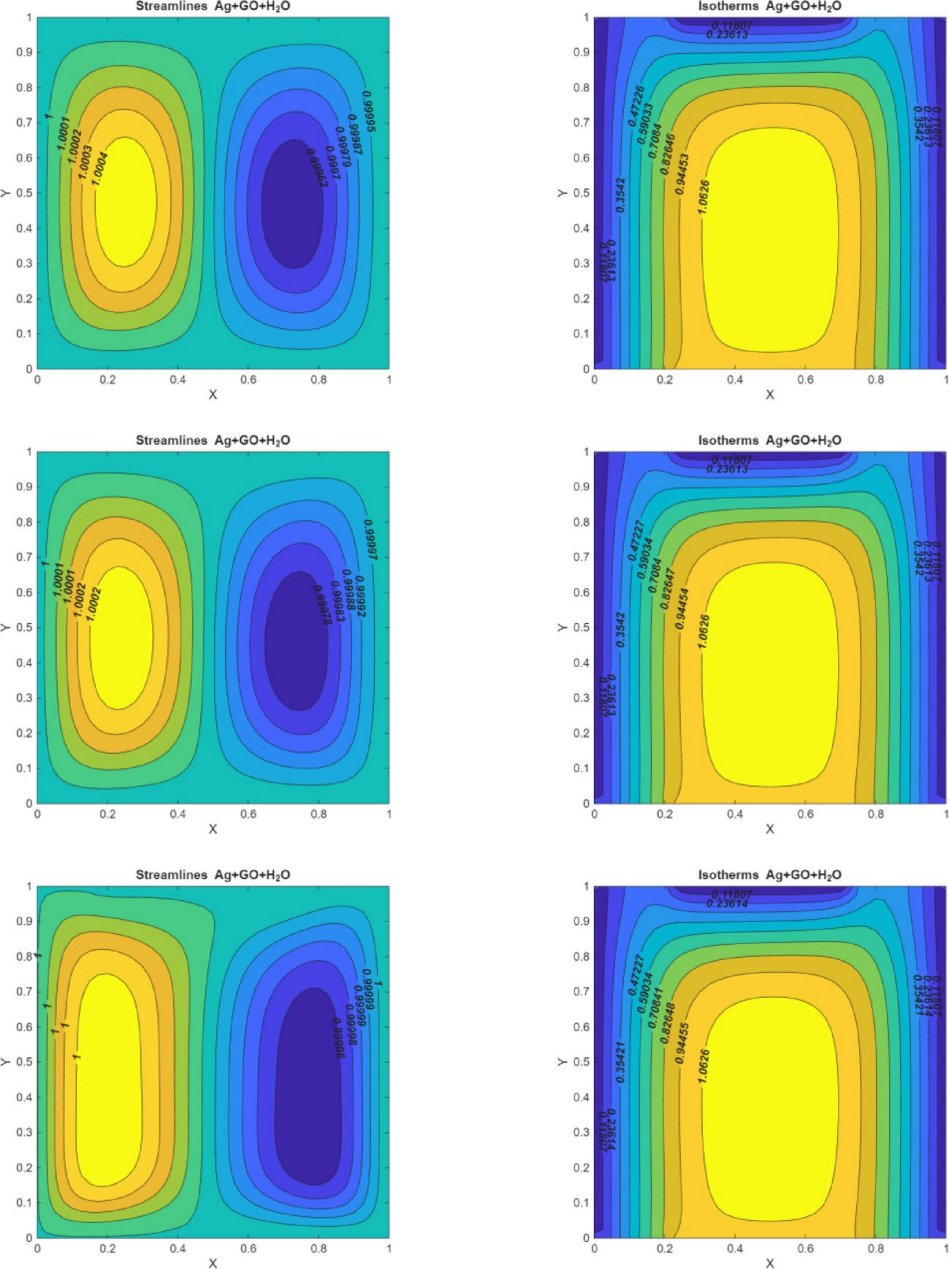
On the left side, figures represent the streamlines for $\:Q=10$, $\:Ri=0.1,\:\:Pr=6.2,\:\:Re=10$; (I)$\:Ha=0$(II) $\:Ha=10$ (III) $\:Ha=50\:.\:\:$On the right side, figures represent the isotherms for $\:Q=10$, $\:Ri=0.1,\:\:Pr=6.2,\:\:Re=10$; (I)$\:Ha=0$(II) $\:Ha=10$ (III) $\:Ha=50$.

Figure 4 (I-III) represents the dominance of developing the magnetic force on the dynamic actions within the enclosure for different values of $\:(Ha=0,\:10,\:50).$ When the magnetic force isn’t present $\:(Ha=0)$ the enclosure contains two circulation vortices that attempt to fill the entire enclosure and heat is concentrated near the left walls. When Ha is raised $\:(Ha=10\:and\:50)$ the curved lines of streamlines appear to be reflected in two profiles, demonstrating that the strength of the curve diminishes as the magnetic field strengthens, which occurs by the Lorentz force. As the magnetic field strength increases to $\:Ha=10$ and $\:Ha=50$, the Lorentz force increasingly hinders the fluid’s movement. This results in smoother streamlines and reduced vortex intensity. At $\:Ha=50$, the fluid’s motion is greatly suppressed, leading to a less dynamic flow and diminished vortex formation. Whereas when implementing the magnetic effect $\:(Ha\:=\:50),$ it is noticed that the fluid motion in the enclosure becomes more diminished than in the condition of $\:Ha\:=\:0$. The fluid’s motion is generally diminished by the impact of magnetic force. The magnetic field is entirely connected to kinetic energy.

According to the isotherms, when the magnetic force is applied, heat distribution changes significantly. The heat tends to spread towards the central regions of the eddies, resulting in colder profiles forming, except near the bottom wall. Additionally, an oval-shaped eddy develops at the center of the enclosure. As the magnetic field strength increases, the magnitude of this central eddy also increases. This observation indicates that the temperature gradient within the enclosure decreases with stronger magnetic fields. This reduction in temperature gradient occurs because the magnetic force impedes fluid movement, limiting rotational flow and thereby affecting the overall heat distribution.

### Reynolds number effects

**Fig. 5:**
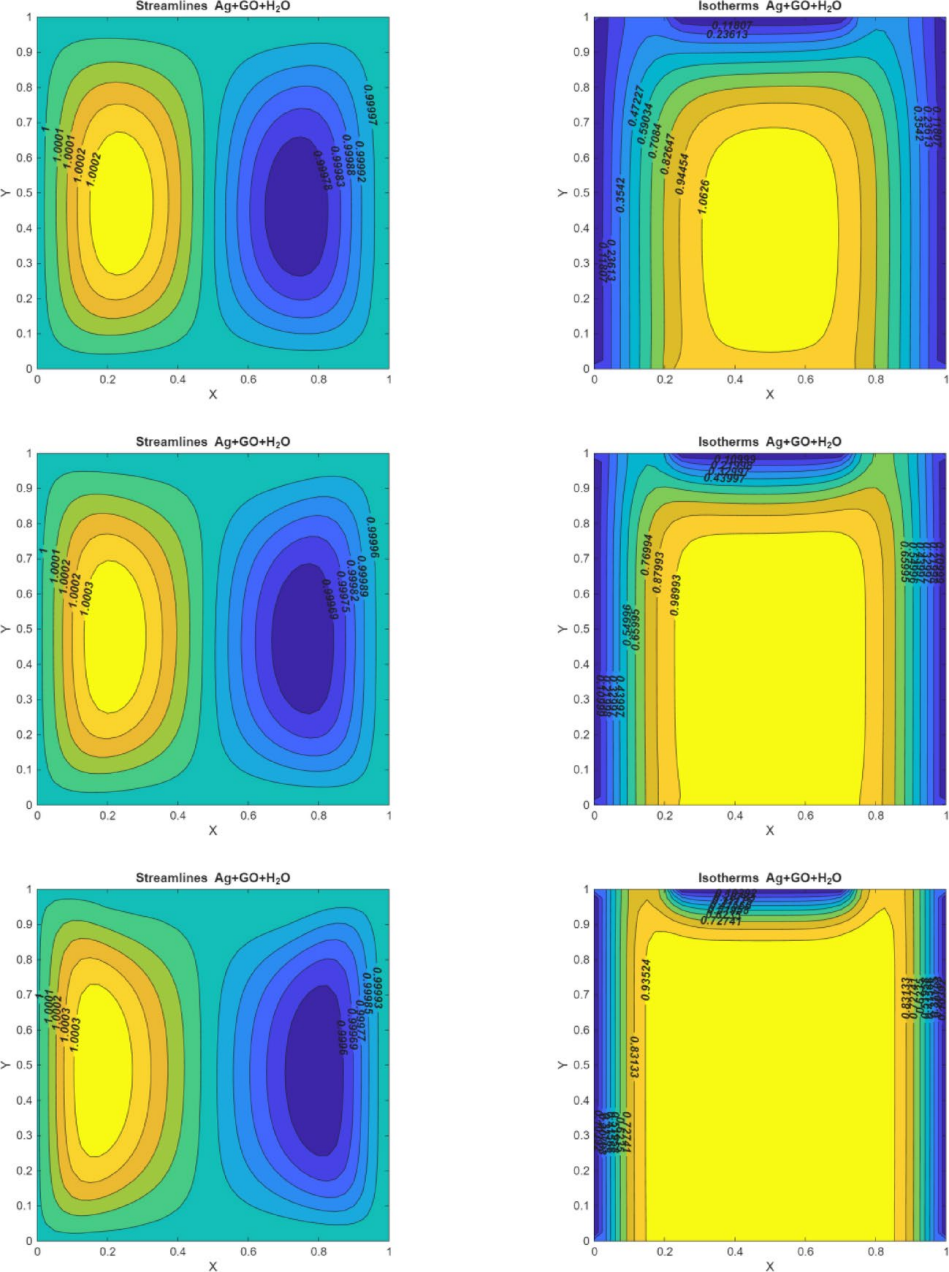
On the left side, figures represent the streamlines for $\:Q=10$, $\:Ri=0.1,\:\:Pr=6.2,\:Ha=10$; (I)$\:Re=10$(II) $\:Re=20$ (III) $\:Re=50.\:\:$On the right side, figures represent the isotherms for $\:Q=10$, $\:Ri=0.1,\:\:Pr=6.2,\:Ha=10$; (I)$\:Re=10$(II) $\:Re=20$ (III) $\:Re=50$.

Figure 5 (I-III) represents the Reynolds number for various values of the streamlines, and isotherms. The Reynolds number displays the proportion of inertial to viscous forces. As $\:Re\:$increases, leading to higher fluid velocity. This acceleration in flow is a direct result of the reduced relative importance of viscous damping compared to inertial effects. In laminar flow, increased velocity causes a more pronounced and streamlined flow pattern, which affects the formation and behavior of circulations within the fluid. The $\:Re$ plays a participating a significant in the transport features of viscous fluids. Raising the $\:Re$ values influences the fluid flow velocity which forms the two circulations. The hot and cold eddies are generated on the left vertical and right vertical sides of the walls. These circulations are generated due to changes in velocity and pressure gradients as the flow adjusts to higher Reynolds numbers. The presence of these circulations reflects the fluid’s response to increasing inertial forces, which dominate over viscous forces. As the Reynolds number increases, these eddies become more defined, reflecting improved heat transfer efficiency and more pronounced temperature gradients.

According to isotherms, when $\:Re=10$ the isotherms at the middle portion are formed in the ovel-shaped hot region. This indicates that, at lower Reynolds numbers, the fluid flow remains relatively laminar, resulting in a more confined hot region. When $\:(Re=20,\:and\:Re=30)$ shows that enhancing the $\:Re$ values cause the flow to be high which enables the temperature to spread more in the oval-shaped field. The formation of hot and cold eddies on the vertical sides of the walls becomes more pronounced. As a result, raising the $\:Re$ values implies enhancing the density profiles so that the fluid flow developed. This is due to the effectiveness of inertial forces over viscous forces.

### Richardson number effects

**Fig. 6:**
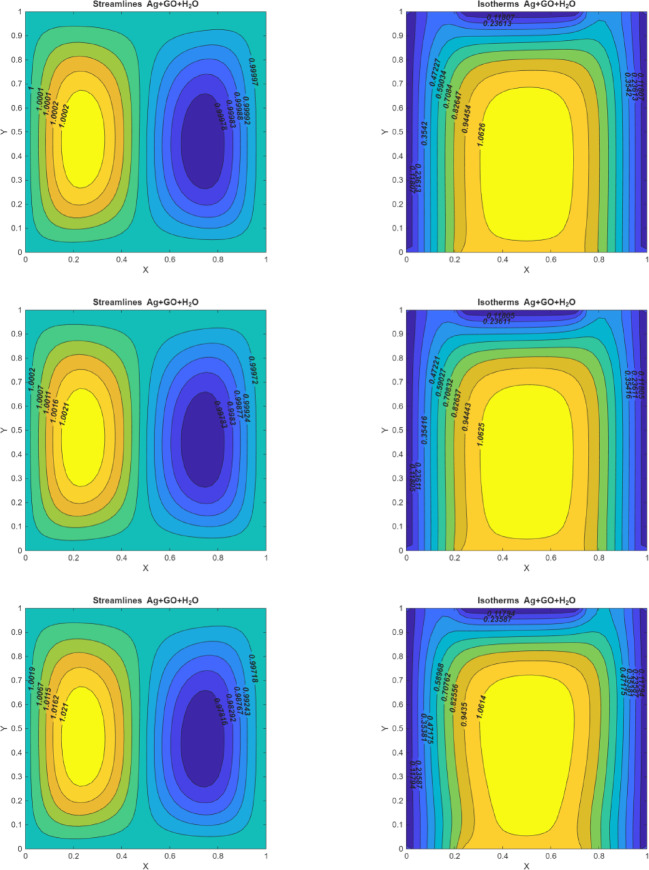
On the left side, figures represent the streamlines for $\:Q=10$, $\:Re=10,\:\:Pr=6.2,\:Ha=10$; (I) $\:Ri=0.1$ (II) $\:Ri=1$ (III) $\:Ri=10.$ On the right side, figures represent the isotherms for $\:Q=10$, $\:Re=10,\:\:Pr=6.2,\:Ha=10$; (I) $\:Ri=0.1$ (II) $\:Ri=1$ (III) $\:Ri=10.$.

Figure 6 (I-III) represents the Richardson number on streamlines and isotherms for three multiple values of $\:(Ri=\text{0.1,1},10)$. The Richardson number plays a main role in determining the type of convection. Three different convection types exist: forced convection $\:(Ri=0.1)$, mixed convection $\:(Ri=1),$ and free convection $\:(Ri=10)$. At $\:Ri\:=\:0.1$, the streamlines are primarily horizontal, indicating that the flow is dominated by external shear forces. With a low Richardson number, buoyancy effects are minimal, leading to a simpler flow pattern with minimal vertical motion. The streamlines are aligned with the direction of forced convection. For mixed convection cases i.e., for $\:Ri=1$ two equal anti-clockwise circulating vortices are noticed, revealed that buoyancy and shear forces possess the same impact on the movement in the enclosure. When the $\:Ri\:$is greater than 1, the free convection circumstance is more optimal because the anti-clockwise circulation cells disappear and the buoyancy force becomes stronger, which causes the shear force and buoyancy forces to interact. The dominance of streamlines also increases at a greater range of the Richardson number, as observed by viewing the size append on the right side of each figure. Additionally, as the Richardson number increases, the isotherms are partially pulled downward and tilted toward the left side vertical wall because of inclination, which indicates greater heat transfer from the left side heat source. At the small value of the Richardson number, the velocity within the chamber will not be a significantly affected by the buoyancy force. The significant influence of buoyancy force is upper during the mixed convection system in place. At higher Richardson numbers $\:(Ri\:>\:1)$, buoyancy forces are considerably stronger within the enclosure, leading to the disappearance of anti-clockwise circulation cells and more dominant free convection. This shift reveals a transition from forced to free convection, emphasizing the growing influence of buoyancy forces as the Richardson number increases. Increasing of Richardson number reveal growth of influence of free convection to forced convection.

### Heat source/sink effects

**Fig. 7:**
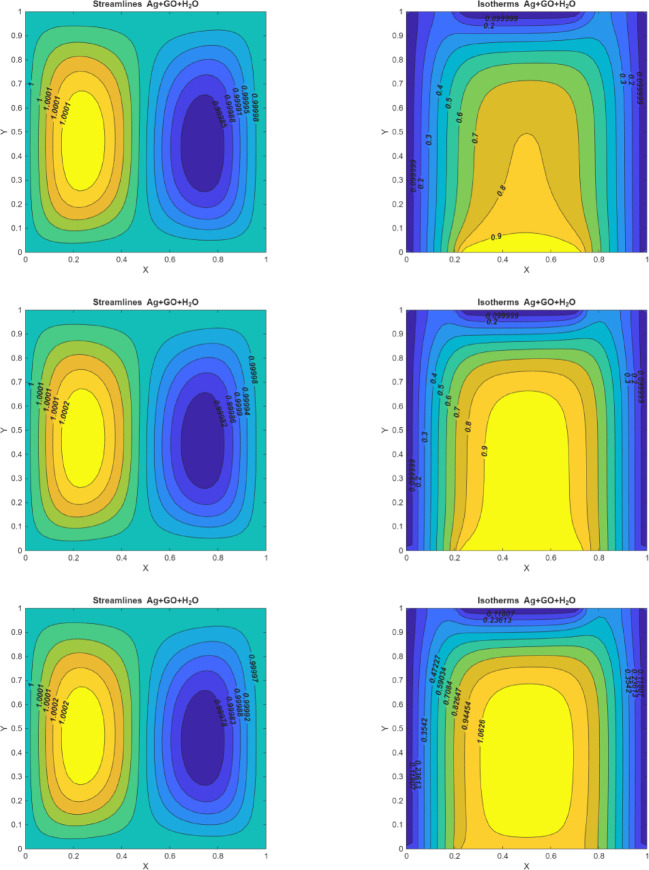
On the left side, figures represent the streamlines for $\:Re=10,\:Pr=6.2,Ri=0.1,\:Ha=10$; (I) $\:Q=-10$ (II)$\:Q=0$ (III)$\:\:\:Q=10$. On the right side, figures represent the isotherms for $\:Q=10$, $\:Re=10,\:\:Pr=6.2,\:Ha=10$; (I) $\:Q=-10$ (II)$\:Q=0$ (III)$\:\:\:Q=10$.

Figure 7 (I-III) represents the heat source / sink on streamlines and isotherms for multiple values of $\:Q$. For three cases $\:(Q=-10,\:\:0,\:10)$ equation metric circulations generated in the eddies. For $\:Q\:=\:-10$, a strong heat sink, the streamlines indicate reduced fluid motion with metric circulations generated in the eddies, demonstrating a decrease in kinetic energy and diminished velocity profiles. In the case $\:(Q=0)$, where no additional heat is added or removed (meaning there is neither a heat source nor a sink), the streamlines illustrate moderate circulatory motion driven solely by temperature gradients imposed at the boundaries. Conversely, for $\:Q\:=\:10$, a heat source, the fluid motion intensifies, leading to higher velocity profiles and increased circulation within the eddies. This indicates that the introduction of heat enhances the buoyancy-driven flow, promoting greater fluid movement. The stronger circulations suggest that the heat source actively promotes buoyant-driven flow, converting thermal energy into kinetic energy and enhancing the overall fluid movement. This intensified circulation amplifies the convective transport, resulting in more dynamic fluid mixing and a vigorous flow pattern.

According to the isotherms, notable changes are observed in the heat sink term. When $\:Q\:=\:-10,$ the isotherms show a concentration of colder temperatures near the center of the enclosure, creating a bell-shaped curve extending from the heat wall side. This suggests that the heat sink effectively extracts thermal energy, leading to a cooler central region. The isotherms under $\:Q=0$ present a symmetric and evenly distributed temperature field, showing a smooth gradient from the heat wall towards the cooler regions. This pattern underscores the absence of external heat perturbations, leading to a stable and consistent convective heat transfer. The isotherm distribution highlights a balanced convective flow where heat is transferred evenly without concentration or deficit, providing a reference for comparison with heat source and sink effects. As the convective heat transfer increases with $\:Q\:=\:10$, more heat transmission occurs in the center part of the enclosure, causing the bell-shaped curve to transform into an oval-shaped eddy. This eddy maintains higher temperatures in the central region while the surrounding areas remain relatively cooler. This indicates that the heat source not only increases thermal energy but also redistributes it more effectively within the enclosure, enhancing overall heat transfer and modifying the temperature distribution patterns.

### Reynolds number characteristics for the Nusselt number distribution

**Fig. 8:**
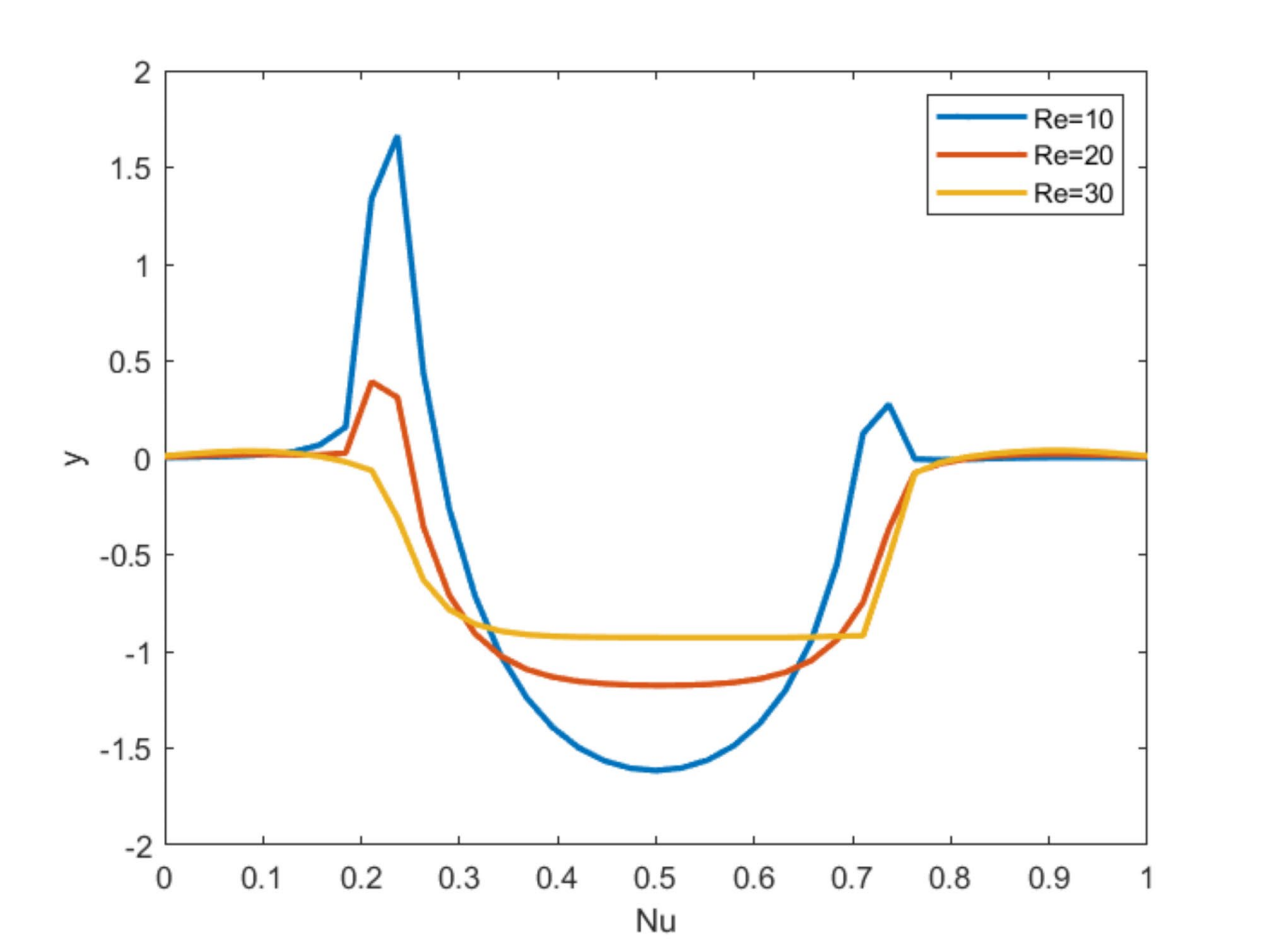
Nusselt number for $\:Pr=6.2, \:Ha=10,\:Q=10$* Ri =* 0.1 $\:Re=10\:,Re=20\:,Re=30.$.

Figure 8 shows Nusselt number $\:\left(Nu\right)$ for multiple values of $\:Re$. Since $\:Nu$ demonstrate the relationship of convective to conductive heat transfer, the value has been viewed that magnetic fields have little effect on $\:Nu$ for low values of $\:Re$ but have a significant impact on $\:Nu$ for high values of $\:Re$, which accelerates convective heat transfer at borders.

### Richardson number characteristics for the Nusselt number distribution

**Fig. 9:**
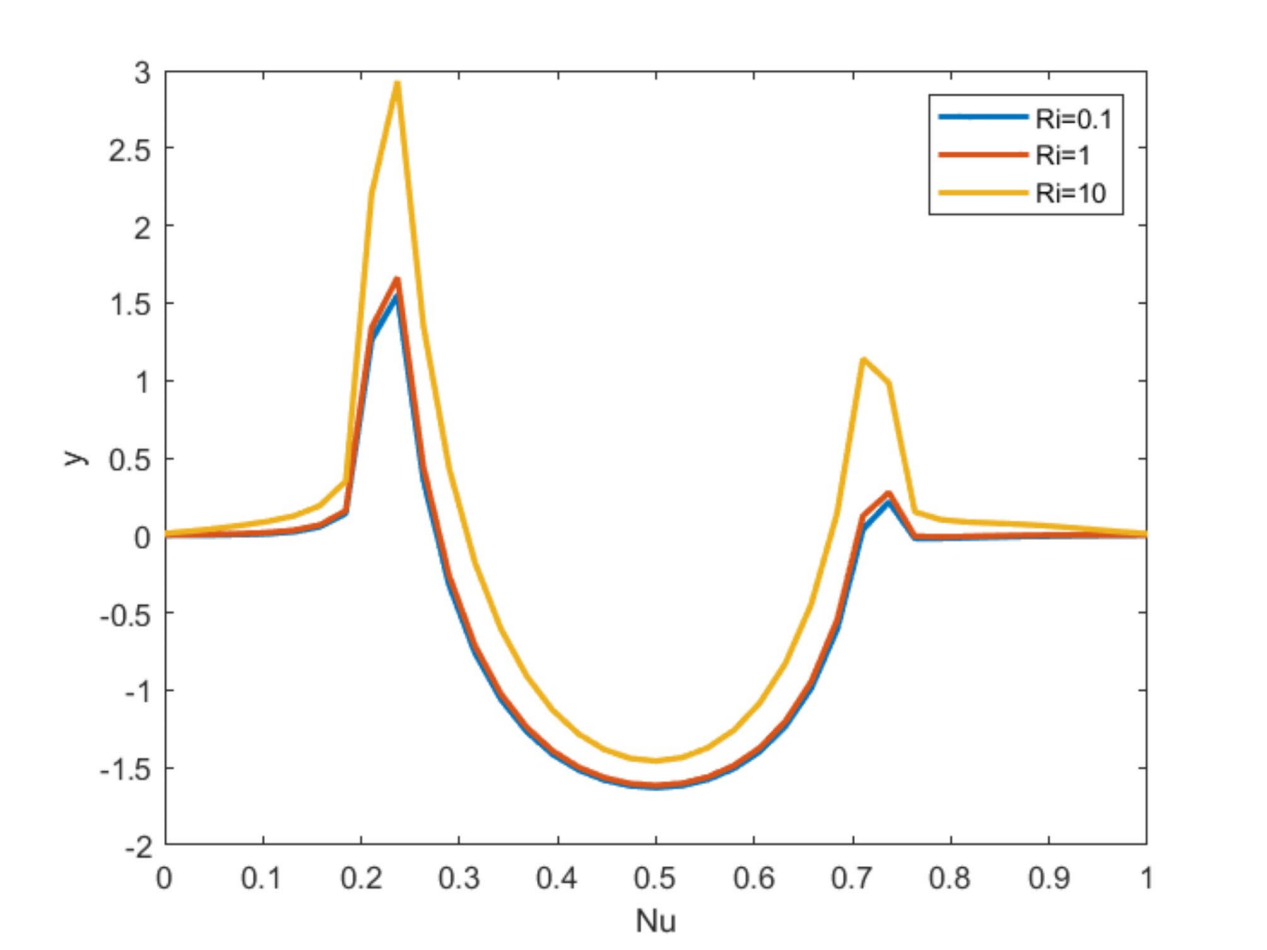
Nusselt number for = 6.2, $\:Ha=10,\:Q=10,\:\:Re=10; \:Ri=0.1 \text{(Blue)}\:Ri=1 \text{(Red)}\:Ri=10 \text{(Orange)}$ .

Figure 9 displays the Nusselt number profiles for the Richardson number for multiple values. As the Richardson number rises, consequently rises the Nusselt number. Also, for raising all $\:Ri$ values the Nusselt number also increases and they coincide as depicted in the figure.

### Properties of Nusselt number distribution for Hartmann number

**Fig. 10:**
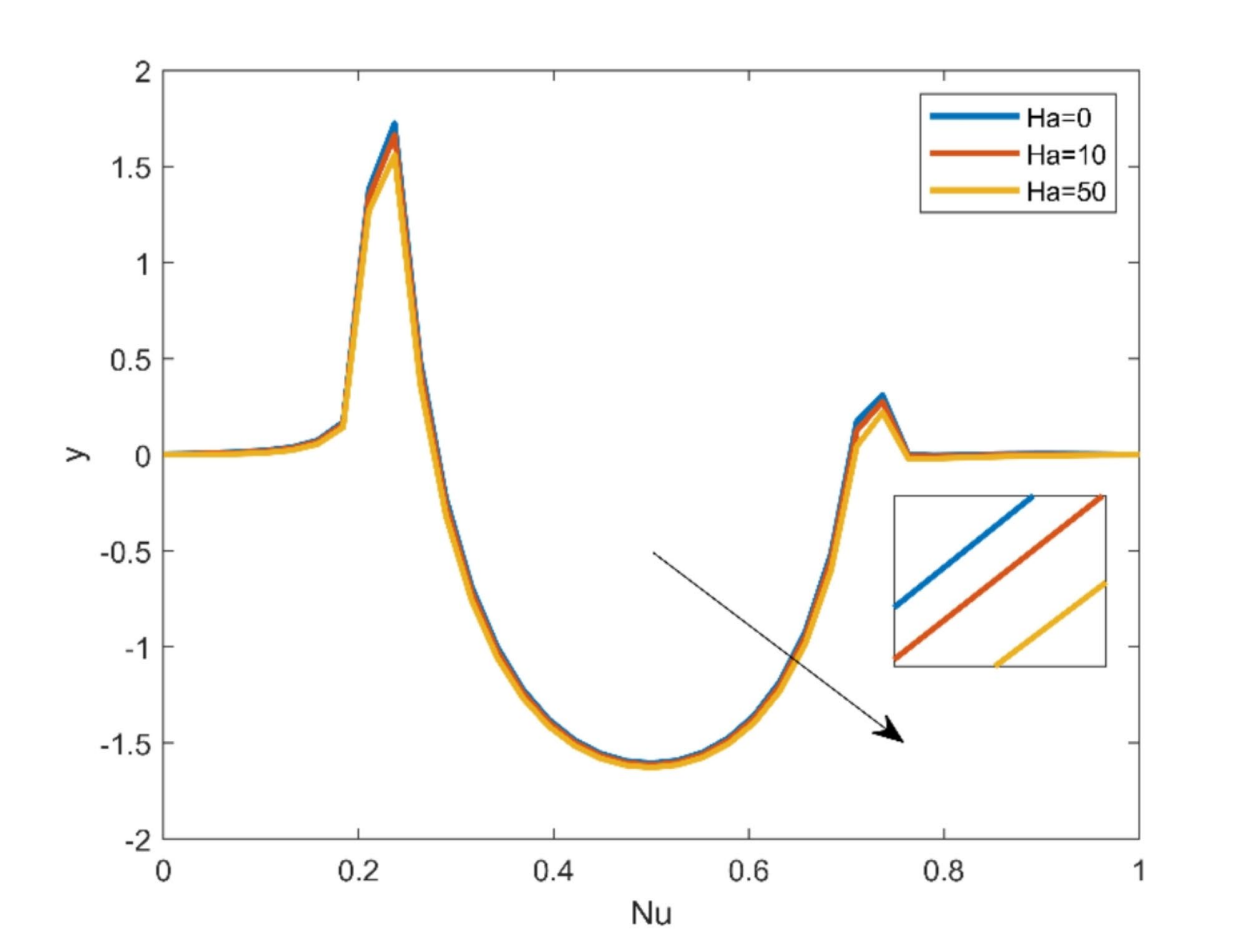
Nusselt number for = 6.2, $\:Ri=0.1,\:Q=10,\:\:Re=10; \:Ha=0\text{(Blue)}\:Ha=10 \text{(Red)}\:Ha=50 \text{(Orange)}$ .

The effect of different Magnetic Parameter values on local heat transfer rate is expressed in Figure 10. The findings shows a slight improvement is noticed for magnifying the Hartmann number values.

## Conclusion

In this article, a numerical computation is executed and exposed to investigate the combined effect of free convection and viscous dissipation in $\:Ag/GO$ -water nanofluids flow in a square cavity, and this is inspired by expanding uses in hybrid fuel cells and techniques for processing magnetic nanoparticles. The top center portion of the chamber remained cool while the bottom center portion remained heated. The right and left vertical walls maintained a cold temperature, with the remaining portion of the cavity being adiabatic. The current study used the MAC Method to numerically simulate the concomitant influence of viscous dissipation and free convection flow of $\:Ag/GO-$water hybrid nanofluids in a square container. The findings of this research are as below:

- The fluid’s temperature and velocity increase inside the square chamber as the solid content of the base fluid increases, indicating enhanced thermal conductivity and heat transfer rates. This highlights the effectiveness of $\:Ag/GO$ hybrid nanofluids in enhancing heat transfer due to their superior thermal properties.
- Increasing the Hartmann number $\:\left(Ha\right)\:$weakens fluid motion and vortex intensity due to the Lorentz force. Higher $\:Ha\:$leads to smoother streamlines and reduced vortex formation. Heat distribution becomes more uniform, with heat spreading towards the center and a larger central eddy forming. The temperature gradient decreases as the magnetic field restricts fluid movement and convection.
- Variations in the Reynolds number significantly impact heat transfer, demonstrating the importance of fluid flow dynamics in thermal management. Higher Reynolds numbers correspond to increased convective heat transfer, which is critical for optimizing the performance of systems using hybrid nanofluids.
- Richardson numbers are crucial for optimizing heat transfer in hybrid nanofluid systems. Low values indicate forced convection, while higher values shift to mixed and free convection, enhancing heat transfer through stronger buoyancy effects. Understanding these dynamics is key to improving thermal management in practical applications.
- Introducing a heat source $\:(Q=10)$ significantly boosts fluid motion and heat transfer, leading to a more uniform temperature distribution within the cavity. Conversely, the presence of a heat sink $\:(Q=-10)$ reduces fluid motion, resulting in a cooler central region and effective management of temperature gradients. These observations provide valuable insights into optimizing thermal systems and underscore the importance of precise control over heat source and sink parameters to achieve efficient heat transfer and improved fluid dynamics.

The findings of the current research demonstrate that the MAC Method is a reliable numerical tool for simulating the fluid flow and heat transfer behaviours of $\:Ag/GO$-water hybrid nanofluids in cavity flow problems. The study provides valuable insights into optimizing thermal management systems in various industrial applications, such as hybrid fuel cells and magnetic nanoparticle processing, through the strategic use of hybrid nanofluids and the manipulation of flow and thermal parameters.

## Data Availability

The datasets generated and/or analysed during the current study are not publicly available due to confidentiality, but are available from the corresponding author on reasonable request.

## Abbreviations

*Gr*: Grashof number
*G*: gravitational acceleration
*P*: Pressure
*Ag*: Silver
*Go*: Graphene
Da: Darcy parameter
*Nu*: Local Nusselt number
*Pr*: Prandtl number
*Re*: Reynold number
*K*: Vortex viscosity parameter
*Ri*: Richardson number
*Q*: Heat flux
*T*: Temperature
*X*: Dimensionless coordinate in the horizontal direction
*U*: Non-dimensional *X*-directional velocity component
*u*: Dimensional *x*-directional velocity component
*x*: Cartesian coordinate in horizondal direction
*Y*: Dimensionless coordinates in the vertical direction
*V*: Non-dimensional *Y*-directional velocity component
*y*: Cartesian coordinate in the vertical direction
*v*: Dimensional *y*-directional velocity component

## Greek letters

*β*: Coefficient of thermal expansion
*α*: Thermal diffusivity
*γ*: Casson fluid parameter
*σ*: Electric conductivity
*θ*: Dimensionless temperature
*φ*: Nanofluid concentration
μ: Dynamic viscosity
*ρhnf*: Hybrid nanofluid Density

## Subscripts

*bf*: Base fluid
*hnf*: Hybrid Nanofluid
*H*: Hot
*C*: Cold

## References

[1] Tayebi, T. & Chamkha, A. J. Entropy generation analysis during MHD natural convection flow of hybrid nanofluid in a square cavity containing a corrugated conducting block. *Int. J. Numer. Methods Heat. Fluid Flow. ***30** (3), 1115–1136. 10.1108/HFF-04-2019-0350 (2020).

[2] Haq, R. U., Soomro, F. A., Mekkaoui, T. & Al-Mdallal, Q. M. MHD natural convection flow enclosure in a corrugated cavity filled with a porous medium. *Int. J. Heat. Mass. Transf. ***121**, 1168–1178. 10.1016/j.ijheatmasstransfer.2018.01.063 (2018).

[3] Rahman, M. M., Pop, I. & Saghir, M. Z. Steady free convection flow within a titled nanofluid saturated porous cavity in the presence of a sloping magnetic field energized by an exothermic chemical reaction administered by Arrhenius kinetics. *Int. J. Heat. Mass. Transf. ***129**, 198–211. 10.1016/j.ijheatmasstransfer.2018.09.105 (2019).

[4] Hussain, S., Ahmad, S., Mehmood, K. & Sagheer, M. Effects of inclination angle on mixed convective nanofluid flow in a double lid-driven cavity with discrete heat sources. *Int. J. Heat. Mass. Transf. ***106**, 847. 10.1016/j.ijheatmasstransfer.2016.10.016 (2017).

[5] Hussain, S., Zeeshan, M. & Sagheer, D. S. Irreversibility analysis for the natural convection of Casson fluid in an inclined porous cavity under the effects of magnetic field and viscous dissipation. *Int. J. Therm. Sci. ***179**, 107699. 10.1016/j.ijthermalsci.2022.107699 (2022).

[6] Ferroudj, N., Koten, H., Kachi, S. & Boudebous, S. Erratum: Prandtl Number Effects on the Entropy Generation During the Transient Mixed Convection in a Square Cavity Heated from Below (Periodica Polytechnica Mechanical Engineering 65:4 (310–325) DOI: 10.3311/PPme.17563), Period. Polytech. Mech. Eng., vol. 65, no. 4, p. 398, 2021, doi: (2021). 10.3311/PPME.19293

[7] Grosan, T., Revnic, C., Pop, I. & Ingham, D. B. Magnetic field and internal heat generation effects on the free convection in a rectangular cavity filled with a porous medium. *Int. J. Heat. Mass. Transf. ***52**, 5–6. 10.1016/j.ijheatmasstransfer.2008.08.011 (2009).

[8] Parthiban, S. & Prasad, V. R. Magneto-convective -Water hybrid nanofluid flow in a heated enclosure containing non-Darcy porous medium: lattice Boltzmann simulation. *Eur. Phys. J. Plus*. **137** (9), 1–20. 10.1140/epjp/s13360-022-03266-6 (2022).

[9] Mehryan, S. A. M., Izadpanahi, E., Ghalambaz, M. & Chamkha, A. J. Mixed convection flow caused by an oscillating cylinder in a square cavity filled with /water hybrid nanofluid. *J. Therm. Anal. Calorim. ***137** (3), 965–982. 10.1007/s10973-019-08012-2 (2019).

[10] Arifuzzaman, M. & Uddin, M. J. Convective flow of alumina-water nanofluid in a square vessel in presence of the exothermic chemical reaction and hydromagnetic field. *Results Eng. ***10, no. **10.1016/j.rineng.2021.100226 (March, 2021).

[11] Izadi, M., Sheremet, M. A. & Mehryan, S. A. M. Natural convection of a hybrid nanofluid affected by an inclined periodic magnetic field within a porous medium, Chinese J. Phys., vol. 65, no. March, pp. 447–458, doi: (2020). 10.1016/j.cjph.2020.03.006

[12] Geridönmez, B. P. & Öztop, H. F. Effects of partial magnetic field in a vented square cavity with aiding and opposing of MWCNT-water nanofluid flows, Eng. Anal. Bound. Elem., vol. 133, no. August, pp. 84–94, doi: (2021). 10.1016/j.enganabound.2021.08.024

[13] Yıldız, C., Arıcı, M. & Karabay, H. Comparison of a theoretical and experimental thermal conductivity model on the heat transfer performance of /water hybrid-nanofluid. *Int. J. Heat. Mass. Transf. ***140**, 598–605. 10.1016/j.ijheatmasstransfer.2019.06.028 (2019).

[14] Kashyap, D. & Dass, A. K. Effect of boundary conditions on heat transfer and entropy generation during two-phase mixed convection hybrid water nanofluid flow in a cavity, Int. J. Mech. Sci., vol. 157–158, no. April, pp. 45–59, doi: (2019). 10.1016/j.ijmecsci.2019.04.014

[15] Ghalambaz, M., Doostani, A., Izadpanahi, E. & Chamkha, A. J. Phase- change heat transfer in a cavity heated from below: the effect of utilizing single or hybrid nanoparticles as additives. *J. Taiwan. Inst. Chem. Eng. ***72**, 104–115. 10.1016/j.jtice.2017.01.010 (2017).

[16] Mehryan, S. A. M., Kashkooli, F. M., Ghalambaz, M. & Chamkha, A. J. Free convection of hybrid water nanofluid in a differentially heated porous cavity. *Adv. Powder Technol. ***28** (9), 2295–2305. 10.1016/j.apt.2017.06.011 (2017).

[17] Sreedevi, P. & Sudarsana Reddy, P. Effect of magnetic field and thermal radiation on natural convection in a square cavity filled with nanoparticles using Tiwari-Das nanofluid model. *Alexandria Eng. J. ***61** (2), 1529–1541. 10.1016/j.aej.2021.06.055 (2022).

[18] Reddy, P. S., Sreedevi, P. & Reddy, V. N. Entropy generation and heat transfer analysis of magnetic nanofluid flow inside a square cavity filled with carbon nanotubes. *Chem. Thermodyn. Therm. Anal. ***6**, 100045. 10.1016/j.ctta.2022.100045 (2022). no. February.

[19] Goswami, K. D., Chattopadhyay, A., Pandit, S. K. & Sheremet, M. A. Transient thermogravitational convection for magneto hybrid nanofluid in a deep cavity with multiple isothermal source-sink pairs, Int. J. Therm. Sci., vol. 173, no. June p. 107376, 2022, doi: (2021). 10.1016/j.ijthermalsci.2021.107376

[20] Armaghani, T. et al. MHD mixed convection of localized heat source/sink in an water hybrid nanofluid in L-shaped cavity. *Alexandria Eng. J. ***60** (3), 2947–2962. 10.1016/j.aej.2021.01.031 (2021).

[21] Priyadharsini, S. & Sivaraj, C. Numerical simulation of thermo- magnetic convection and entropy production in a ferrofluid filled square chamber with effects of heat generating solid body. *Int. Commun. Heat. Mass. Transf. ***131**, 105753. 10.1016/j.icheatmasstransfer.2021.105753 (2022).

[22] Miroshnichenko, I. V., Sheremet, M. A., Oztop, H. F., Abu-, N. & Hamdeh Natural convection of nanofluid in an open inclined cavity with a heat-generating element. *Int. J. Heat. Mass. Transf. ***126**, 184–191. 10.1016/j.ijheatmasstransfer.2018.05.146 (2018).

[23] Bondarenko, D. S., Sheremet, M. A., Oztop, H. F. & Ali, M. E. Natural convection of nanofluid in a cavity with a heat-generating element. *Heatline visualization Int. J. Heat. Mass. Transf. ***130**, 564–574. 10.1016/j.ijheatmasstransfer.2018.10.091 (2019).

[24] Mansour, M. A., Siddiqa, S., Gorla, R. S. R. & Rashad, A. M. Effects of heat source and sink on entropy generation and MHD natural convection of /water hybrid nanofluid filled with square porous cavity, Therm. Sci. Eng. Prog., vol. 6, no. October pp. 57–71, 2018, doi: (2017). 10.1016/j.tsep.2017.10.014

[25] Selimefendigil, F., Öztop, H. F. & Chamkha, A. J. MHD mixed convection and entropy generation of nanofluid filled lid driven cavity under the influence of inclined magnetic fields imposed to its upper and lower diagonal triangular domains. *J. Magn. Magn. Mater. ***406**, 266–281. 10.1016/j.jmmm.2016.01.039 (2016).

[26] Fazuruddin, S., Sreekanth, S. & Raju, G. S. S. Effect of various tilted positions of a thin fin on natural convection of laminar viscous flow in a square cavity. *Int. J. Heat. Technol. ***39** (5), 1634–1642. 10.18280/IJHT.390527 (2021).

[27] Vinodhini, N. & Prasad, V. R. NUMERICAL study of MAGNETO convective Buongiorno nanofluid flow in a rectangular enclosure under oblique magnetic field with heat generation/absorption and complex wall conditions, Heliyon, vol. 9, no. 7, p. e17669, doi: (2023). 10.1016/j.heliyon.2023.e17669 10.1016/j.heliyon.2023.e17669 PMC10359772 37483737

[28] Prasad, R. & NUMERICAL STUDY OF FREE CONVECTION Ag- WATER NANOFLUID FLOW IN A SQUARE ENCLOSURE WITH VISCOUS DISSIPATION AND HEAT GENERATION/ABSORPTION EFFECTS IN A POROUS MEDIUM WITH COMPLE,. *J. Porous Media*, **26**, 9, 77–99, doi: 10.1615/jpormedia.2023044454. (2023).

[29] Sumithra, A. & Sivaraj, R. Chemically reactive magnetohydrodynamic mixed convective nanofluid flow inside a square porous enclosure with viscous dissipation and ohmic heating. *Eur. Phys. J. Plus*. **137** (10). 10.1140/epjp/s13360-022-03409-9 (2022).

[30] Basak, T. & Chamkha, A. J. International Journal of Heat and Mass transfer heatline analysis on natural convection for nanofluids confined within square cavities with various thermal boundary conditions. *Int. J. Heat. Mass. Transf. ***55**, 21–22. 10.1016/j.ijheatmasstransfer.2012.05.025 (2012).

[31] Rashidi, I., Mahian, O., Lorenzini, G., Biserni, C. & Wongwises, S. International Journal of Heat and Mass Transfer Natural convection of water nanofluid in a square cavity: Effects of heterogeneous heating. *Int. J. Heat. Mass. Transf. ***74**, 391–402. 10.1016/j.ijheatmasstransfer.2014.03.030 (2014).

[32] Rath, S. International Journal of Heat and Mass transfer complex interplay of power-law rheology and non-ob erb eck-boussinesq effects on natural convection heat transfer in a confined domain. *Int. J. Heat. Mass. Transf. ***176**, 121462. 10.1016/j.ijheatmasstransfer.2021.121462 (2021).

[33] Teamah, M. A. & El-Maghlany, W. M. Augmentation of natural convective heat transfer in square cavity by utilizing nanofluids in the presence of magnetic field and uniform heat generation/absorption. *Int. J. Therm. Sci. ***58**, 130–142. 10.1016/j.ijthermalsci.2012.02.029 (2012).

[34] Revnic, C., Grosan, T., Pop, I. & Ingham, D. B. Magnetic field effect on the unsteady free convection flow in a square cavity filled with a porous medium with a constant heat generation. *Int. J. Heat. Mass. Transf. ***54**, 9–10. 10.1016/j.ijheatmasstransfer.2011.01.020 (2011).

[35] Basak, T., Roy, S. & Balakrishnan, A. R. Effects of thermal boundary conditions on natural convection flows within a square cavity. *Int. J. Heat. Mass. Transf. ***49**, 23–24. 10.1016/j.ijheatmasstransfer.2006.05.015 (2006).

[36] Algehyne, E. A. et al. Analysis of the MHD partially ionized GO-Ag/water and GO-Ag/kerosene oil hybrid nanofluids flow over a stretching surface with Cattaneo-Christov double diffusion model: A comparative study, Int. Commun. Heat Mass Transf., vol. 136, no. June, p. 106205, doi: (2022). 10.1016/j.icheatmasstransfer.2022.106205

[37] Grosan, T., Revnic, C., Pop, I. & Ingham, D. B. Free convection heat transfer in a square cavity filled with a porous medium saturated by a nanofluid. *Int. J. Heat Mass Transf. ***87**, 36–41 (2015).

[38] Ahmad, F. et al. The improved thermal efficiency of Maxwell hybrid nanofluid comprising of graphene oxide plus silver / kerosene oil over stretching sheet. *Case Stud. Therm. Eng. ***27**, 101257. 10.1016/j.csite.2021.101257 (2021).

[39] Ghadikolaei, S. S. & Gholinia, M. 3D mixed convection MHD flow of hybrid nanoparticles in hybrid base fluid under the effect of bond, Int. Commun. Heat Mass Transf., vol. 110, no. December p. 104371, 2020, doi: (2019). 10.1016/j.icheatmasstransfer.2019.104371

[40] Reddy, P. S., Sreedevi, P. & Reddy, V. N. Entropy generation and heat transfer analysis of magnetic nanofluid flow inside a square cavity filled with carbon nanotubes. *Chem. Thermodyn. Therm. Anal. ***6**, 100045. 10.1016/j.ctta.2022.100045 (2022).

[41] Zheng, K. et al. New fractional approach for the simulation of (Ag) and mixed hybrid nanofluid flowing through a channel: Fractal fractional derivative. *Case Stud. Therm. Eng. ***45, no. **10.1016/j.csite.2023.102948 (February, 2023).

[42] Ghasemi, B., Aminossadati, S. M. & Raisi, A. Magnetic field effect on natural convection in a nanofluid-filled square enclosure. *Int. J. Therm. Sci. ***50** (9), 1748–1756. 10.1016/j.ijthermalsci.2011.04.010 (2011).

[43] Rajarathinam, M., Akermi, M., Khan, M. I. & Nithyadevi, N. MHD mixed convection heat transfer of copper water nanofluid in an inclined porous cavity having isothermal solid block. *J. Magn. Magn. Mater. ***593** (171845). 10.1016/j.jmmm.2024.171845 (2024).

[44] Hashemi-Tilehnoee, M., Dogonchi, A. S., Seyyedi, S. M., Chamkha, A. J. & Ganji, D. D. Magnetohydrodynamic natural convection and entropy generation analyses inside a nanofluid-filled incinerator-shaped porous cavity with wavy heater block. *J. Therm. Anal. Calorim. ***141** (5), 2033–2045. 10.1007/s10973-019-09220-6 (2020).

[45] Dogonchi, A. S., Chamkha, A. J. & Ganji, D. D. A numerical investigation of magneto-hydrodynamic natural convection of Cu–water nanofluid in a wavy cavity using CVFEM. *J. Therm. Anal. Calorim. ***135** (4), 2599–2611. 10.1007/s10973-018-7339-z (2019).

[46] Seyyedi, S. M., Dogonchi, A. S., Hashemi-Tilehnoee, M., Ganji, D. D. & Chamkha, A. J. Second law analysis of magneto-natural convection in a nanofluid filled wavy-hexagonal porous enclosure. *Int. J. Numer. Methods Heat. Fluid Flow. ***30** (11), 4811–4836 (2020).

[47] Chamkha, A. J., Hussain, S. H. & Abd-Amer, Q. R. Mixed convection heat transfer of Air inside a Square Vented Cavity with a heated Horizontal Square Cylinder. *Numer. Heat. Transf. Part. A: Appl. ***59** (1), 58–79. 10.1080/10407782.2011.541216 (2011).

[48] Kumar, M., Kaswan, P. & Kumari, M. Entropy generation analysis of microrotating Casson’s nanofluid with Darcy–forchheimer porous media using a neural computing based on Levenberg–Marquardt algorithm. *Int. J. Numer. Methods Heat. Fluid Flow. ***34 No**, 2285–2320. 10.1108/HFF-10-2023-0612 (2024).

[49] Kaswan, P., Kumar, M. & Kumari, M. Analysis of a bioconvection flow of magnetocross nanofluid containing gyrotactic microorganisms with activation energy using an artificial neural network scheme. *Results Eng. ***17**, 101015. 10.1016/j.rineng.2023.101015 (2023).

[50] Agrawal, R. & Kaswan, P. Investigation of the heat performance for squeezed hybrid nanofluid flow between parallel disks embedded in porous medium with thermal radiation. *J. Porous Media*. **25** (8). 10.1615/JPorMedia.2022041525 (2022).

[51] Kumar, M., Kaswan, P., Kumari, M., Ahmad, H. & Askar, S. Cattano Christov double diffusion model for third grade nanofluid flow over a stretching Riga plate with entropy generation analysis. *Heliyon*. **10 **10.1016/j.heliyon.2024.e30188 (2024). 10.1016/j.heliyon.2024.e30188 PMC11128471 38803878

[52] Kumar, M., Kaswan, P. & Kumari, M. Numerical simulation of entropy generation analysis of MHD hybrid-nanofluid flow with nonlinear thermal radiation and melting heat transfer. *Special Top. Reviews Porous Media: Int. J. ***13** (6). 10.1615/SpecialTopicsRevPorousMedia.2022044874 (2022).

