# Correction: Numerical study of magneto convective ag (silver) graphene oxide (GO) hybrid nanofluid in a square enclosure with hot and cold slits and internal heat generation/absorption

**DOI:** 10.1038/s41598-025-24903-x

**Published:** 2025-10-20

**Authors:** Elayaraja Rajenderan, V. Ramachandra Prasad

**Affiliations:** Department of Mathematics, School of Advances Sciences, Vellore Institute of Technology, Vellore, 632014 India

Correction to: *Scientific Reports* 10.1038/s41598-024-76233-z, published online 22 October 2024

The original Article contained errors. Due to an error in the density of water listed in Table 1, calculations underlying the results of Figures 4 - 7 were incorrect. In addition, Table 1 contained a typographical error in the value for the electrical conductivity of Silver. The density of water has been corrected to 998.2 kgm^–3^.

The original Table 1 and accompanying legend appear below.

**Table 1:** Thermophysical properties of $GO$, $\:Ag$ and $\:{H}_{2}O$^36,38,39^ &^41^.

Property	H_2_O	*Ag* (Silver)	Graphene Oxide (GO)
$\:\rho\:(\text{k}\text{g}{m}^{-3}$)	997.1	10,500	1800
$\:{C}_{p}(\text{J}{kg}^{-1}{K}^{-1})$	4179	235	717
k(W$\:{m}^{-1}{k}^{-1}$)	0.613	429	5000
$\:\beta\:({k}^{-1}$)	21 × 10^−5^	1.89 × 10^−5^	2.84 × 10^−4^
$\:\sigma\:\left({{\Omega\:}}^{-1}{m}^{-1}\right)$	0.05	63 × 10^−6^	6.30 × 10^7^
$\:\mu\:\left(Pa.\:s\right)$	8.9 × 10^−4^	–	–
Pr	6.2	–	–

The original Figures 4, 5, 6, 7 and their corresponding legends appear below.

**Fig. 4:**
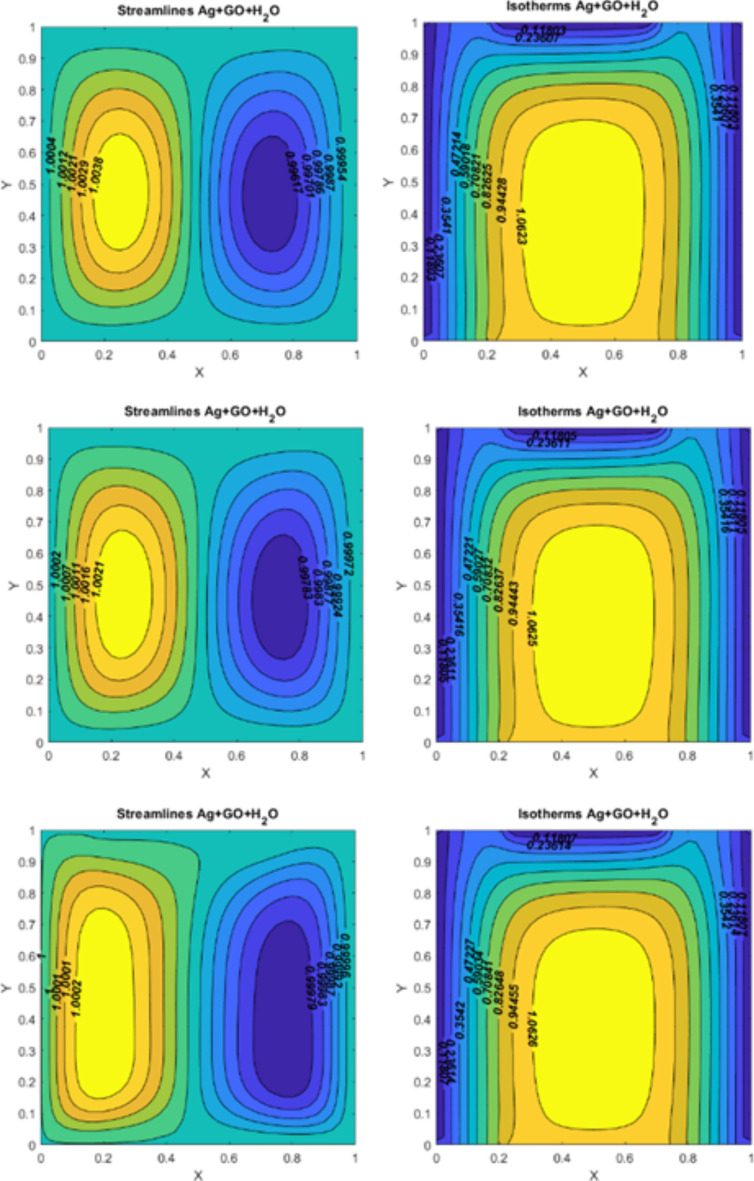
On the left side, figures represent the streamlines for $\:Q=10$, $\:Ri=0.1,\:\:Pr=6.2,\:\:Re=10$; (I)$\:Ha=0$ (II) $\:Ha=10$ (III) $\:Ha=50\:.\:\:$On the right side, figures represent the isotherms for $\:Q=10$, $\:Ri=0.1,\:\:Pr=6.2,\:\:Re=10$; (I)$\:Ha=0$ (II) $\:Ha=10$ (III) $\:Ha=50$.

**Fig. 5:**
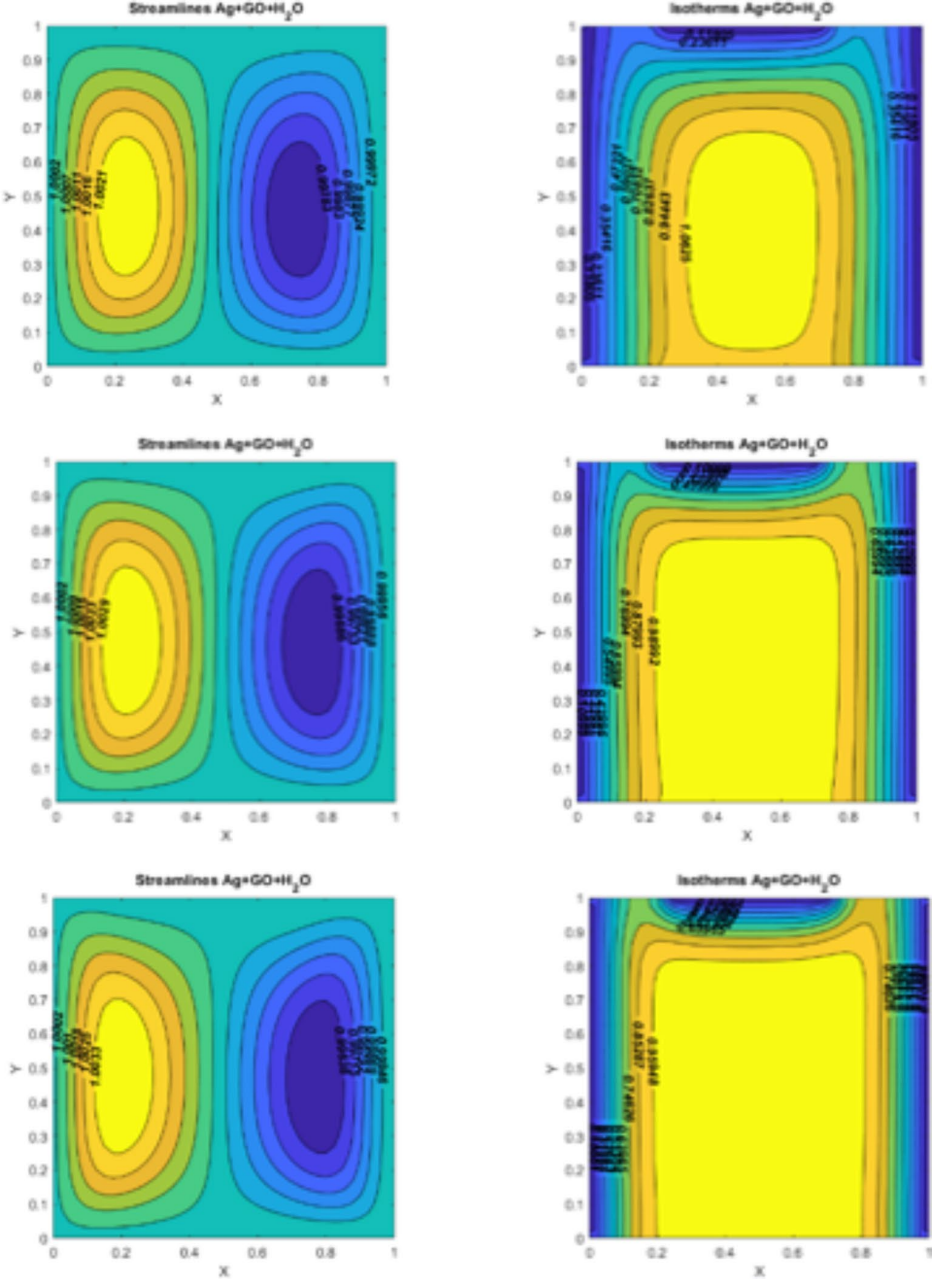
On the left side, figures represent the streamlines for $\:Q=10$, $\:Ri=0.1,\:\:Pr=6.2,\:Ha=10$; (I)$\:Re=10$ (II) $\:Re=20$ (III) $\:Re=50.\:\:$On the right side, figures represent the isotherms for $\:Q=10$, $\:Ri=0.1,\:\:Pr=6.2,\:Ha=10$; (I)$\:Re=10$ (II) $\:Re=20$ (III) $\:Re=50$.

**Fig. 6:**
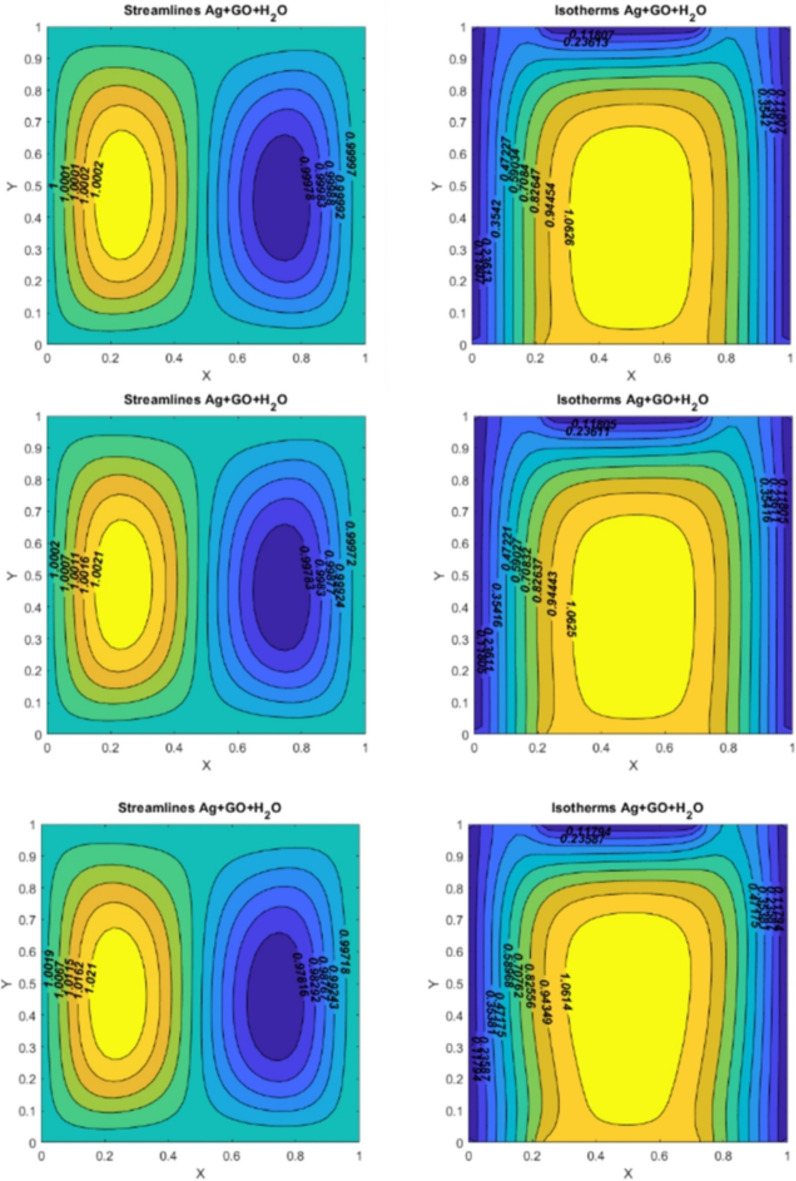
On the left side, figures represent the streamlines for $\:Q=10$, $\:Re=10,\:\:Pr=6.2,\:Ha=10$; (I) $\:Ri=0.1$ (II) $\:Ri=1$ (III) $\:Ri=10.$ On the right side, figures represent the isotherms for $\:Q=10$, $\:Re=10,\:\:Pr=6.2,\:Ha=10$; (I) $\:Ri=0.1$ (II) $\:Ri=1$ (III) $\:Ri=10$.

**Fig. 7:**
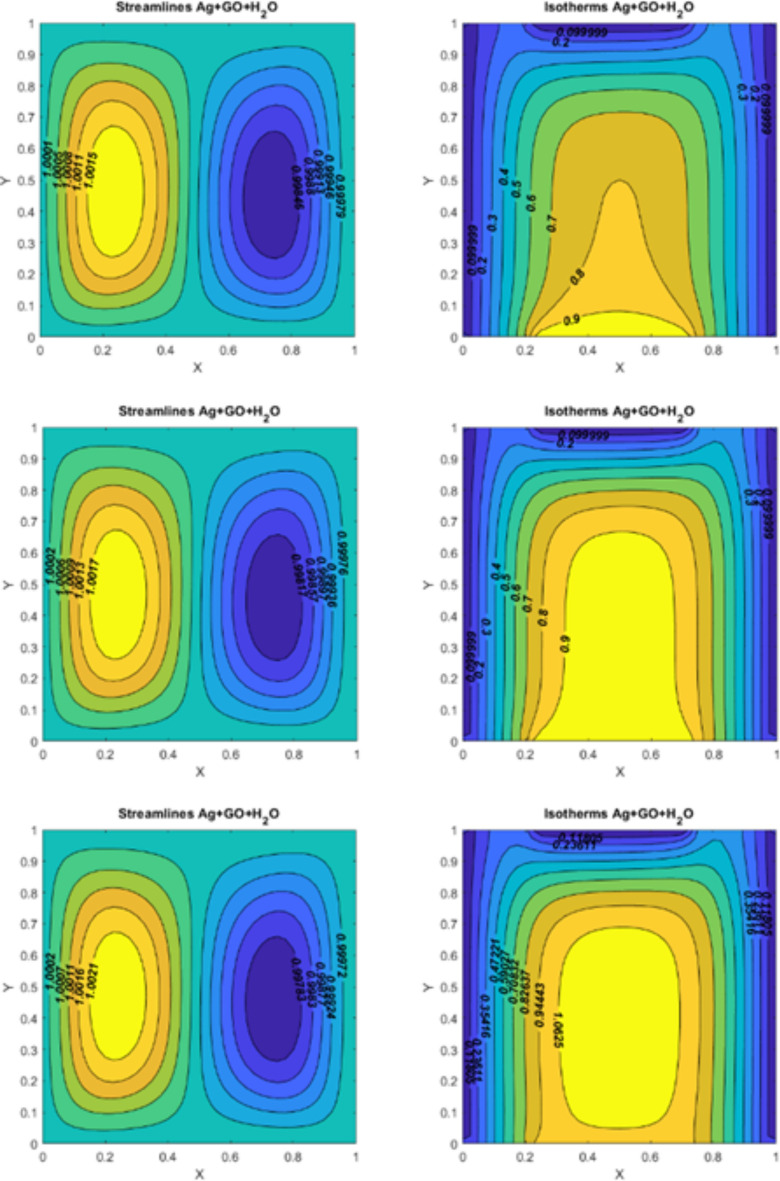
On the left side, figures represent the streamlines for $\:Re=10,\:Pr=6.2,Ri=0.1,\:Ha=10$; (I) $\:Q=-10$ (II)$\:Q=0$ (III)$\:\:\:Q=10$. On the right side, figures represent the isotherms for $\:Q=10$, $\:Re=10,\:\:Pr=6.2,\:Ha=10$; (I) $\:Q=-10$ (II)$\:Q=0$ (III)$\:\:\:Q=10$.

The original Article has been corrected.

